# Photophysical properties of cationic dyes captured in the mesoscale channels of micron-sized metal-organic framework crystals

**DOI:** 10.1038/s41598-018-28080-y

**Published:** 2018-06-29

**Authors:** In-Hwan Choi, Suk Bin Yoon, Seong Huh, Sung-Jin Kim, Youngmee Kim

**Affiliations:** 1 Department of Chemistry and Protein Research Center for Bio-Industry, Hankuk University of Foreign Studies, Yongin, 17035 Republic of Korea; 2 Institute of Nano-Bio Technology and Department of Chemistry and Nano Science, Ewha Womans University, Seoul, 03760 Republic of Korea

## Abstract

The optical properties of dye molecules in confined spaces can differ from the solution phase due to confinement effects. Pre-organized mesoscale channels of metal-organic frameworks (MOFs) are very suited for hosting various dyes, and the robust frameworks often render the encapsulated dyes with certain preferential geometries, which are different from those found in solution. Furthermore, pre-organized open channels can efficiently guide the uniform and unique spatial distribution of dye molecules in a controlled manner, which are otherwise difficult to achieve. Thus, sufficiently large dye molecules can avoid the formation of complex aggregates when captured inside open channels. In contrast, small dye molecules can form well-defined dimers or aggregates. The resulting dye-encapsulated MOFs can display unusual photophysical properties of the captured dyes. An anionic framework of In-BTB with mesoscale 3D channels is utilized for the efficient encapsulation of various cationic dyes through cation-exchange processes. Six different cationic dyes are encapsulated in the anionic framework of In-BTB, and their crystal structures are completely solved. Novel photophysical properties of these spatially distributed dye molecules in dye@In-BTBs are investigated.

## Introduction

The unique physicochemical properties of metal-organic frameworks (MOFs) primarily stem from either their metal-based nodes or polytopic organic bridging ligands with a wide range of functionalities^1–3^. Additionally, MOFs usually possess high surfaces and large pore volumes and thereby are suitable for various advanced applications^4–6^. Selective CO_2_ capture^7^ at moderate conditions and high storage ability of energy-related gases, such as H_2_ and CH_4_, have accelerated the research for new types of crystalline hybrid porous materials^8^. Additionally, MOFs are good heterogeneous catalysts as well as superior catalyst supports for a number of organic transformations and electrocatalysis^9^.

Meanwhile, MOFs can also be utilized for encapsulating various functional guest molecules or ions with high efficacy^10^. Drug delivery systems based on MOFs encapsulating drugs have increasingly attracted attention^11–13^. The robust and tailorable open channels of MOFs can effectively host various biologically active drug molecules for biomedical purposes due to their large voids. Likewise, various dye molecules have also been successfully encapsulated inside MOF channels to visibly demonstrate the large potential voids of MOFs. For instance, high-surface-area MOF-177, [Zn_4_O(BTB)_2_] where BTB = 1,3,5-benzenetribenzoate, can encapsulate 16 molecules of Astrazon Orange R (AOR), 2 molecules of Nile red (NR), and 1 molecule of Reichardt’s dye (RD) per unit cell^14^. The physical dimensions of these dyes increase in the order of AOR < NR < RD. Notably, dye@MOF-177 exhibited the characteristic color of each dye because MOF-177 is colorless. There are numerous examples of reported dye encapsulation by MOFs^15–21^. In most cases, however, it is not clear where the guest molecules are placed inside the void spaces of MOFs due to lack of crystallographic information of the dye-encapsulated MOFs (dye@MOFs). In addition, the mechanistic information of the encapsulation process is not well investigated. Although the colors of MOFs encapsulating specific dye molecules display the characteristic colors of the dyes, there is not much information about the penetration depth of dye molecules into the channels of MOFs. Obviously, once the MOF crystals are placed into the solution of the guest molecules, these molecules start to diffuse into the available channels of the MOF crystals from the outermost parts of the single crystal. Detailed information of the penetration depths for dyes will shed light on the understanding of mechanisms for molecular sieving effects, catalysis and drug delivery.

In this regard, we have been investigating the crystal structures of dye@MOFs. The optical properties of dyes in solution strongly depend on their environment, such as the solvent. In contrast, the lack of detailed crystallographic information of dyes encapsulated in MOFs often hampers the systematic correlation between the structures of dyes and their optical properties. Thus, the spatial positioning of dye molecules inside pre-organized channels of MOFs is an ideal method to investigate the structure-dependent optical properties of dyes. For example, we recently demonstrated that large RD was effectively encapsulated in mesoscale channels of anionic In-BTB, [Et_2_NH_2_]_3_[In_3_(BTB)_4_], through a cation-exchange process^22^. The anionic framework of In-BTB is an especially good host for RD cations despite its large molecular dimensions compared to small neutral acridine orange (AO). The encapsulation amount of AO by In-BTB is much smaller than RD under the same encapsulation conditions. Furthermore, In-BTB containing RD, RD@In-BTB, exhibited distinct visible absorption properties compared to non-encapsulated free RD based on solid-state UV/Vis absorption spectroscopy. To elucidate these different optical properties, the crystal structure of RD@In-BTB was successfully refined for the first time^22^. The structural comparison of the encapsulated RD and the free RD crystal grown in solution showed large distortion of the main molecular axis of RD in RD@In-BTB. This severe distortion is mainly responsible for the weak light absorption in the visible region due to less effective intramolecular charge transfer. This example implies that the photoluminescence (PL) properties of luminescent dyes captured inside pre-organized MOF channels can also be effectively altered due to isolation, confinement, and unique spatial ordering of dye molecules inside mesoscale channels.

**Figure 1:**
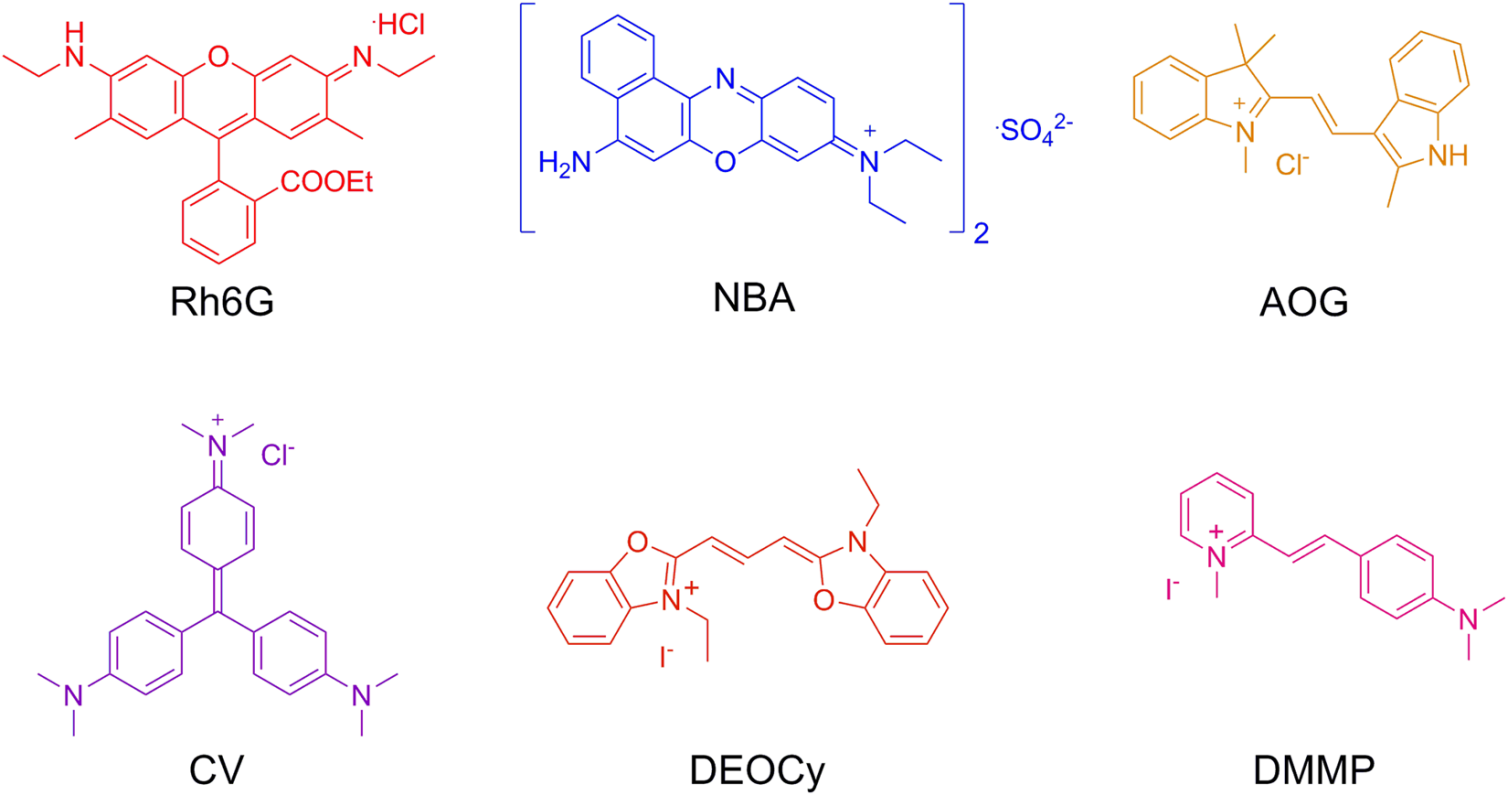
Cationic dyes. Chemical structures of the six cationic dyes for encapsulation.

Motivated from these interesting findings, we attempted to further elucidate the relationship between the positional parameters and/or geometry of a series of cationic dyes with varying dimensions and functionalities encapsulated in the anionic framework of In-BTB and the corresponding PL properties of the captured dyes. In general, In-MOFs containing mononuclear pseudotetrahedral [In(OOCR)_4_]^−^ motifs as nodes of infinite 3D networks possess anionic frameworks with periodically arranged counter-cations located near the anionic In^III^ centers^18,22–27^. The resulting anionic nodes of In-MOFs would be beneficial for encapsulating a range of functional cationic guests within the frameworks through a simple cation-exchange process because of favorable host-guest electrostatic interactions. Notwithstanding, the guest encapsulation abilities of In-MOFs in solution have been rarely studied in detail compared to their gas sorption properties^22,23,27^. In order to explore the encapsulation of guests by an In-MOF in solution, we carefully chose six different cationic dyes for encapsulation, as shown in Fig. 1. The cationic dyes include rhodamine 6 G (Rh6G), nile blue A (NBA), Astrazon Orange G (AOG), crystal violet (CV), 3,3′-diethyloxacarbocyaine iodide (DEOCy), and 2-[4-dimethylamino]styryl)-1-methylpyridinium (DMMP). Although they are monovalent cations, their molecular dimensions and shapes are different from each other. We completely solved the crystal structures of a series of dye@In-BTBs, and their PL properties were investigated.

## Results and Discussion

### Dye encapsulation and crystal structures

Single crystals of In-BTB were prepared by the reaction between In(NO_3_)_3_ hydrate and 1,3,5-benzenetribenzoic acid (H_3_BTB) in DEF based on our literature method^22^. The crystals are formulated as [Et_2_NH_2_]_3_[In_3_(BTB)_4_]·10DEF·14H_2_O. The overall structure is best described as a 2-fold interpenetrated 3D network (Fig. S1). On the basis of PLATON analysis, cation- and solvent-free In-BTB possesses a 71.0% void volume^28^. The corresponding Connolly surfaces clearly demonstrate the large void space of cation- and solvent-free In-BTB (Fig. S2). The real available void should be marginally smaller than the expected value due to counter-cations (Et_2_NH_2_^+^) that were not successfully refined. Six representative colored guest molecules (Rh6G, NBA, AOG, CV, DEOCy, and DMMP) were chosen based on their physical dimensions and charges to systematically investigate the encapsulation properties of the micron-sized as-prepared In-BTB crystals. The large void of In-BTB may be sufficient to capture large cationic dyes, as the center-to-center distances on each BTB^3−^ linker are as large as 15.486(1) Å and 27.034(1) Å alternatively. The dimensions of the free dye molecules are approximately 10.49 × 7.02 Å^2^ for Rh6G, 11.84 × 4.69 Å^2^ for NBA, 11.68 × 4.88 Å^2^ for AOG, 12.25 × 10.77 Å^2^ for CV, 13.19 × 5.70 Å^2^ for DEOCy, and 11.47 × 3.79 Å^2^ for DMMP. The as-prepared In-BTB (10 mg) was immersed into an ethanol solution (10 mL) of each dye with a concentration of 2 mM for 7 d, except for CV and AOG. Saturated ethanol solutions of CV and AOG were used instead because of their low solubility.

**Figure 2:**
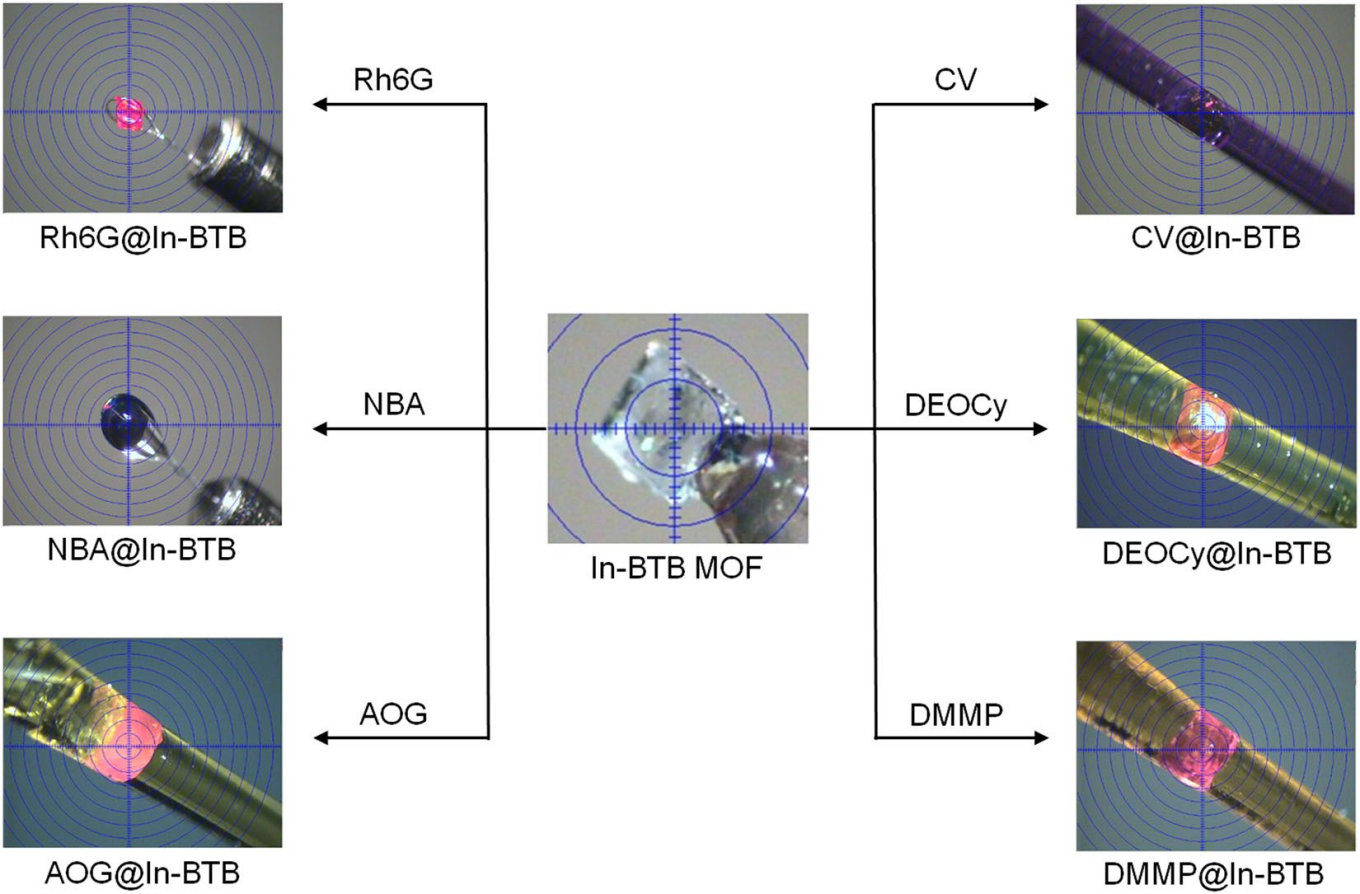
Digital photographs of single crystals. Color-changed crystal images of the as-prepared In-BTB and six dye@In-BTB MOFs. All six dyes were encapsulated into In-BTB in ethanol at room temperature.

All six dye-encapsulating crystals changed color, as shown in Fig. 2, and the color change clearly suggests the efficient encapsulation of the dye molecules. The original colorless In-BTB crystals changed their color into the corresponding dye colors (dye@In-BTB, dye = Rh6G, red; NBA, dark blue; AOG, orange; CV, violet; DEOCy, orange; and DMMP, orange). All dye@In-BTBs maintained crystalline frameworks as evidenced by powder X-ray diffraction patterns (PXRD) as shown in Fig. S3. The diffraction intensities of all six dye@In-BTBs were found to be less intense than the as-prepared In-BTB partly because of low sample amounts for PXRD measurements. Solid-state diffuse reflectance UV/Vis spectra of dye@In-BTBs depicted in Fig. S4 indicate the successful encapsulation of dyes. The six dye@In-BTBs showed a wide range of absorption bands from 200–600 nm containing characteristic absorption band of each dyes. The absorption observed in the UV region can be partly attributed to In-BTB frameworks. Interestingly, the captured NBA and CV cations showed much enhanced absorption in longer wavelength region (600–750 nm) despite no absorption by free dyes in ethanol. FT-IR spectra of dye@In-BTBs are shown in Fig. S5.

**Figure 3:**
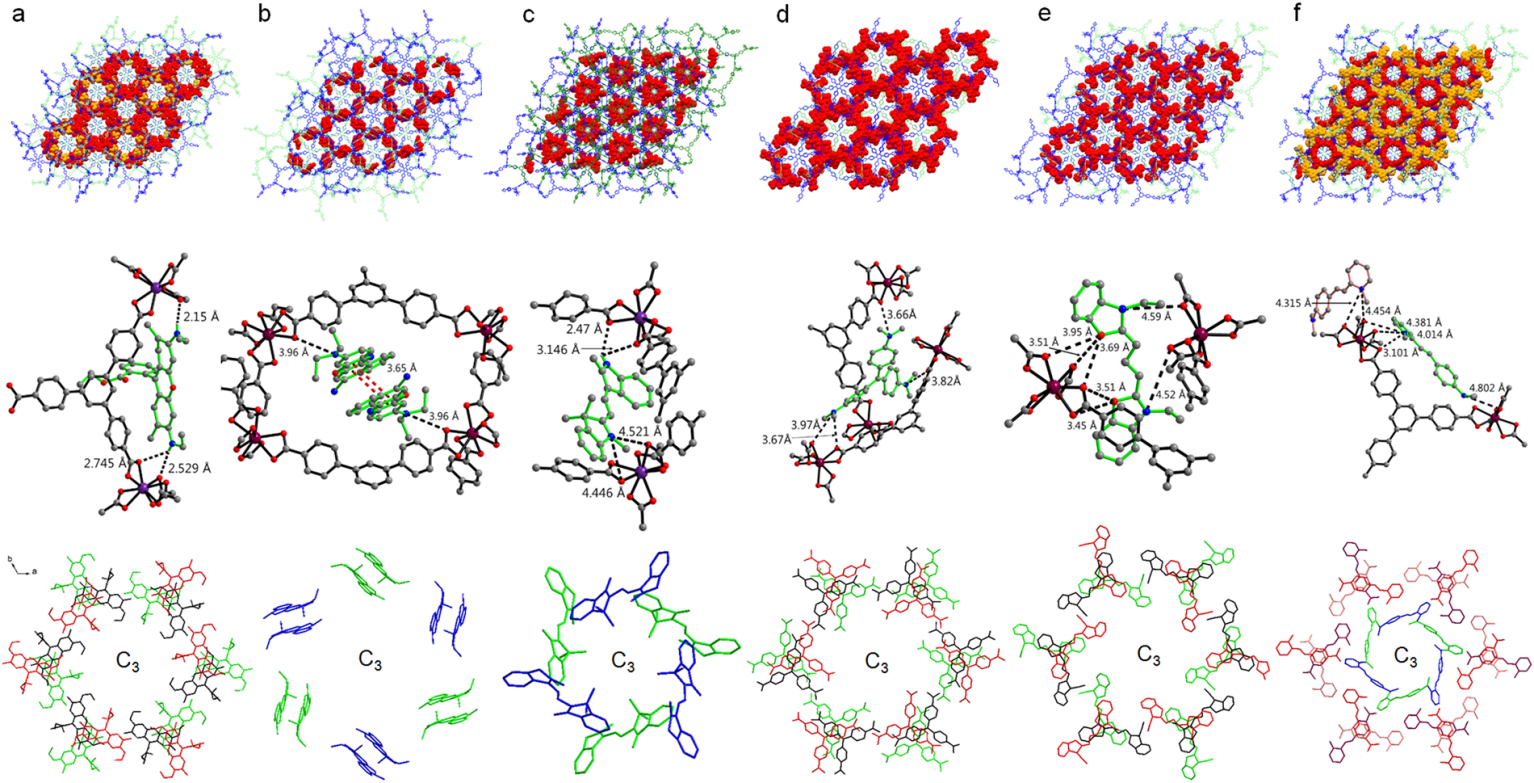
Crystal structures of dye@In-BTB, interactions between dyes and frameworks, and symmetries of dyes. (**a**) Rh6G cation, (**b**) NBA cation, (**c**) AOG cation, (**d**) CV cation, (**e**) DEOCy cation, and (**f**) DMMP cation. Encapsulated dye cations are shown with a CPK model, and the 2-fold interpenetrated frameworks are displayed with a stick model using different colors: blue and green. Three partially occupied dye molecules placed in different layers with *C*_3_ symmetry are represented by different colors. All hydrogen atoms were removed for clarity. Electrostatic interactions between partially occupied dye cations (green bonds) are displayed as black dotted lines. A cationic nitrogen atom and the anionic part of the framework are shown as a blue sphere and black bonds, respectively. Hydrogen bonds (black dotted lines) between the carboxylate O atoms (red) and partially occupied dye N(blue)-H are shown. The π-π interactions between NBA (red dotted lines) are shown.

To investigate the interactions between In-BTB and the dye cations, we analyzed the single-crystal structures of each dye@In-BTB. The occupancies of all the dye cation atoms were fixed to obtain the best fit with the largest residual peaks (Table 1), but the actual occupancies were estimated by other measurements, such as UV/Vis absorption and ^1^H-NMR spectroscopy, which will be discussed in a later section. The 3-fold rotation axis exists in the center of BTB^3−^ ligands alternatively along the *c*-axis in Rh6G@In-BTB (Fig. 3a). Partially occupied Rh6G cations were efficiently confined in the framework through hydrogen bonding (N-H···O_carboxylate_ 2.15–2.745 Å) with *C*_3_ symmetry along the *c*-axis (Fig. 3a). Rh6G layers are placed in a three-layer sequence (ABCABC) along the *c*-axis. Each layer is represented by a different color. The unit cell dimensions of Rh6G@In-BTB deviated by +0.4425 Å on the *a*- and *b*-axes and −1.0419 Å on the *c*-axis from the dimensions of as-prepared In-BTB (Tables 1 and S1). Considering the fact that the as-prepared In-BTB contains both counter-cations and solvent molecules in its mesoscale channels, the guest encapsulation process will replace these cations and solvent molecules with cationic dyes. The xanthene ring of the Rh6G cation is nearly planar with a tilted phenyl ring at an angle 86.7° due to steric hindrance. This tilting angle is much larger than in (Rh6G)_2_[MnCl_4_]·(EtOH)_0.5_^29^ and (Rh6G)_2_[CdCl_4_]·(EtOH)·(H_2_O)^30^ with tilting angles of 77.5° and 79.75°, respectively. The carbonyl group is located near the xanthene ring, and it is the most likely configuration, as determined from previous theory^31^.

**Table 1:** Crystal data of In-BTB and dye-encapsulated In-BTBs.

	In-BTB^*a*^	RD@In-BTB^*a*^	Rh6G@In-BTB	NBA@In-BTB	AOG@In-BTB	CV@In-BTB	DEOCy@In-BTB	DMMP@In-BTB
Empirical formula	C_72_H_36_In_2_O_16_	C_185_H_75_In_4_NO_34_	[C_72_H_24_In_2_O_16_][C_28_H_22_N_2_O_3_]_0.25_[C_3_HNO]_0.25_	[C_72_H_31_In_2_O_16_][C_20_H_17_N_3_O]_0.25_	[C_72_H_27_In_2_O_16_][C_22_H_11_N_2_]_0.25_	[C_72_H_31_In_2_O_16_][C_25_H_12_N_3_]_0.25_	[C_72_H_31_In_2_O_16_][C_21_H_15_N_2_]_0.25_	[C_72_H_28_In_2_O_16_][C_16_H_13_N_2_]_0.25_[C_16_H_10_N_2_]_0.25_
Formula weight	1386.65	3314.74	1499.99	0.71073	1453.40	1470.14	1463.42	1494.47
Temp. (K)	296(2)	295(2)	223(2)	223(2)	296(2)	296(2)	296(2)	296(2)
Wavelength (Å)	0.71073	0.71073	1.54178	1.54178	0.71073	0.71073	0.71073	0.71073
Space group	R-3	R-3	R-3	R-3	R-3	R-3	R-3	R-3
a (Å)	44.2269(19)	45.597(6)	44.6694(10)	45.0162(8)	44.8184(13)	45.2799(19)	44.8708(7)	44.9141(10)
b (Å)	44.2269(19)	45.597(6)	44.6694(10)	45.0162(8)	44.8184(13)	45.2799(19)	44.8708(7)	44.9141(10)
c (Å)	42.519(2)	40.718(8)	41.4771(11)	41.8896(9)	42.1828(14)	41.588(2)	42.0747(7)	41.9851(10)
Volume (Å^3^)	72026(6)	73314(21)	71673(4)	73515(3)	73380(5)	73843(7)	73363(3)	73348(4)
Z	18	9	18	18	18	18	18	18
Crystal size (mm)	0.43 × 0.40 × 0.21	0.20 × 0.20 × 0.12	0.16 × 0.14 × 0.10	0.16 × 0.15 × 0.10	0.22 × 0.22 × 0.18	0.25 × 0.25 × 0.20	0.28 × 0.20 × 0.20	0.28 × 0.28 × 0.20
Goodness-of-fit on F^2^	0.869	0.671	1.345	0.962	0.923	1.050	0.995	0.967
Final R indices [I > 2σ(I)]	R_1_ = 0.0608 wR_2_ = 0.1598	R_1_ = 0.0925 wR_2_ = 0.2502	R_1_ = 0.1749 wR_2_ = 0.4290	R_1_ = 0.0709, wR_2_ = 0.2128	R_1_ = 0.1232 wR_2_ = 0.2551	R_1_ = 0.1539 wR_2_ = 0.3515	R_1_ = 0.0824 wR_2_ = 0.2301	R_1_ = 0.0944 wR_2_ = 0.2556
CCDC number	976960	988834	1533264	1533265	1533175	1533176	1533235	1534603

^a^See ref.^22^.

Partially occupied pairs of NBA cations are located in the framework with *C*_3_ symmetry alternatively along the *c*-axis (Fig. 3b). There are possible electrostatic interactions between the anionic framework and NBA cations. Two symmetric NBA cations are paired interacting with each other through π-π interactions with a separation distance of 3.65 Å, as shown in Fig. 3b. The relatively small size of the NBA cation may be responsible for the effective formation of pairs inside the channels. The unit cell dimensions of NBA@In-BTB deviated by +0.4329 Å on the *a*- and *b*-axes and −0.5204 Å on the *c*-axis from the dimensions of as-prepared In-BTB (Tables 1 and S1).

The encapsulation pattern of partially occupied AOG cations is similar to that observed in NBA@In-BTB through N-H···O_carboxylate_ hydrogen bonding and electrostatic interactions with *C*_3_ symmetry alternatively along *c*-axis (Fig. 3c). Upon encapsulation of AOG, the unit cell dimensions of AOG@In-BTB deviated by +0.5915 Å on the *a*- and *b*-axes and −0.3362 Å on the *c*-axis from the original values of In-BTB (Tables 1 and S1).

In the cases of CV and DEOCy, partially occupied cations are located in the framework in a similar pattern to that of Rh6G through possible electrostatic interactions with *C*_3_ symmetry along the *c*-axis (Fig. 3d,e). As for Rh6G@In-BTB, the CV or DEOCy layers are placed in a three-layer sequence (ABCABC) along the *c*-axis. The unit cell dimensions of CV@In-BTB and DEOCy@In-BTB deviated by +1.053 Å and + 0.6439 Å on the *a*- and *b*-axes and −0.939 Å and −0.4443 Å on the *c*-axis from the original values of In-BTB, respectively (Tables 1 and S1). The three dihedral angles formed from the three phenyl rings in the CV cation are 122.14°, 126.03°, and 129.78°, which are comparable to the average dihedral angle of 125° formed from in [CV][BPh_4_]^32^.

In contrast to other dye@In-BTBs, two crystallographic independent DMMP cations were found for DMMP@In-BTB. They were captured possibly through electrostatic interactions with *C*_3_ symmetry alternatively along the *c*-axis for one inner DMMP and *C*_3_ symmetry along the *c*-axis for the other outer DMMP (Fig. 3f). The inner DMMP cations were encapsulated in a similar pattern to those observed in NBA@In-BTB and AOG@In-BTB, and the outer cations were encapsulated in similar pattern to those observed in Rh6G@In-BTB, CV@In-BTB and DEOCy@In-BTB. The unit cell dimensions of DMMP@In-BTB deviated by +0.6872 Å on the *a*- and *b*-axes and −0.5339 Å on the *c*-axis from the original values of In-BTB (Tables 1 and S1). These results may imply that the other five dyes can be encapsulated in the framework in similar patterns to DMMP@In-BTB, as we could refine only some of encapsulated dye cations due to the high disorder of the dyes (SQUEEZE/PLATON was used in the X-ray structural refinement). Interestingly, the structure of the DMMP cation in the framework has a *trans* configuration, and the pyridinium and phenyl rings are tilted with a tilting angle of 24.33°, which is considerably different from that in free [DMMP][I]^33^. This more tilted structure might be originated from the perfect fitting into the geometry of pre-organized mesoscale channels as well as electrostatic interactions between the DMMP cations and the anionic framework.

The cation- and solvent-free dye@In-BTBs contain smaller void volumes than the void (71.0%) of the cation- and solvent-free In-BTB based on PLATON analysis (52.7% for Rh6G, 61.4% for NBA, 61.2% for AOG, 59.9% for CV, 61.9% for DEOCy, and 55.7% for DMMP). These indicate that the dyes were encapsulated by 25.8% for Rh6G, 13.5% for NBA, 13.8% for AOG, 15.6% for CV, 12.8% for DEOCy, and 21.5% for DMMP of the original void volume (71.0%) of In-BTB.

### Imaging of Rh6G in Rh6G@In-BTB

**Figure 4:**
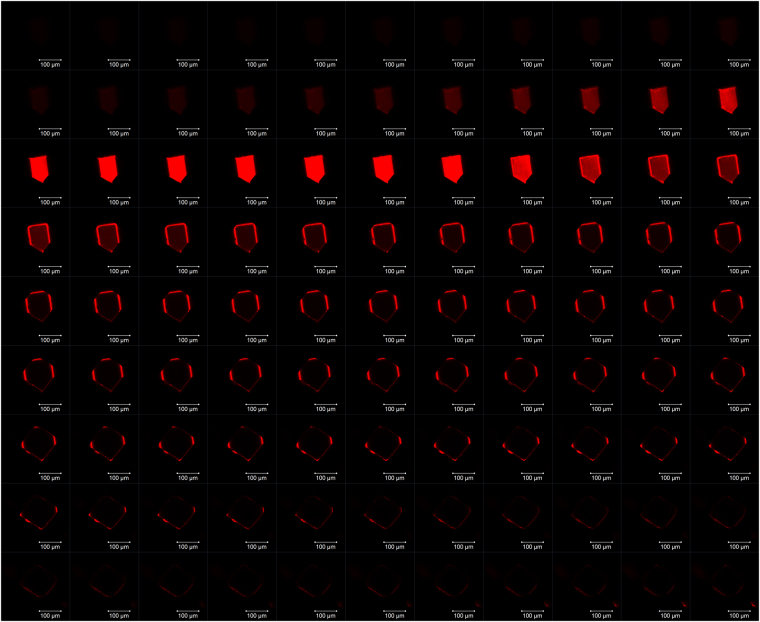
CLSM image. Z-stack fluorescence CLSM images of the Rh6G@In-BTB crystal (λ_ex_ = 488 nm, λ_em_ = 518 nm). The slicing distance is 1.25 μm. Scale bars represent 100 μm.

Fluorescence confocal laser scanning microscopy (CLSM) of a single crystal of Rh6G@In-BTB was performed to check the distribution and penetration depth of Rh6G cations. The morphologies of single crystals of In-BTB were analyzed by scanning electron microscopy (SEM) to acquire crystal morphology information before performing the CLSM investigation. The crystals have two seemingly different morphologies. Relatively large crystals exhibited more complex shapes with pentagonal crystal facets, while small crystals showed truncated cubic shapes (Fig. S6). However, both crystals showed the same crystal structure and unit cell dimensions in the X-ray diffraction study. The two morphologies were observed even in the same batch of samples. All dye@In-BTBs exhibited similar crystal morphologies (Fig. S7). The Z-stack CLSM images depicted in Fig. 4 show that the Rh6G ions did not fully penetrate the internal part of the In-BTB crystal. They are mainly located on the peripheral parts of the crystal, as evidenced by the characteristic red fluorescence of the Rh6G cations. Thus, the cation exchange of [Et_2_NH_2_]^+^ counter-cations with Rh6G cations mostly occurs at the outermost parts of the crystal. This can be accounted for by the fact that the initial cation exchange near the crystal surfaces gradually blocks the available channels to reduce the channel dimensions because the large Rh6G cations can be relatively strongly held near the anionic In^III^ nodes through electrostatic interaction. Therefore, additional Rh6G cations cannot be easily encapsulated further after a certain period of time. It can also be inferred from the series of Z-stack CLSM images in Fig. 4 that the crystal placed on the glass slide displayed a flat pentagonal crystal facet with a uniform distribution of Rh6G cations, while the top surface was not parallelly oriented relative to the bottom flat pentagonal facet. The Rh6G penetration depths are in the range of 5 ~ 13 μm based on the sliced CLSM image (Fig. S8). Nevertheless, it is quite interesting to note that even incomplete penetration of the Rh6G cations into the internal parts of the In-BTB crystal gave sufficient diffraction data for refinement of the structure.

### Fluorescence spectroscopic analysis

Rh6G is a well-known cationic xanthene dye used in dye lasers due to its high quantum yield (QY)^34^. Therefore, its PL properties are of significance in many optical applications; however, Rh6G has a problem of forming molecular aggregates. Rh6G encapsulated in a solid matrix, such as silica gel, usually displays uncontrollable aggregation, which often strongly affects its emission properties^35^. De Camargo and coworkers studied means to reduce dye aggregation. The dye aggregation is believed to occur through π-π interactions of the aromatic rings. To prevent aggregation, they used mesoporous sol-gel hosts. De Camargo *et al*. mentioned that to minimize aggregation effects, the most favorable host matrices are those in which the anionic binding sites are diluted and highly dispersed^34^. Thus, MOFs with anionic frameworks are good candidates to satisfy these two conditions to prevent dye aggregation.

**Figure 5:**
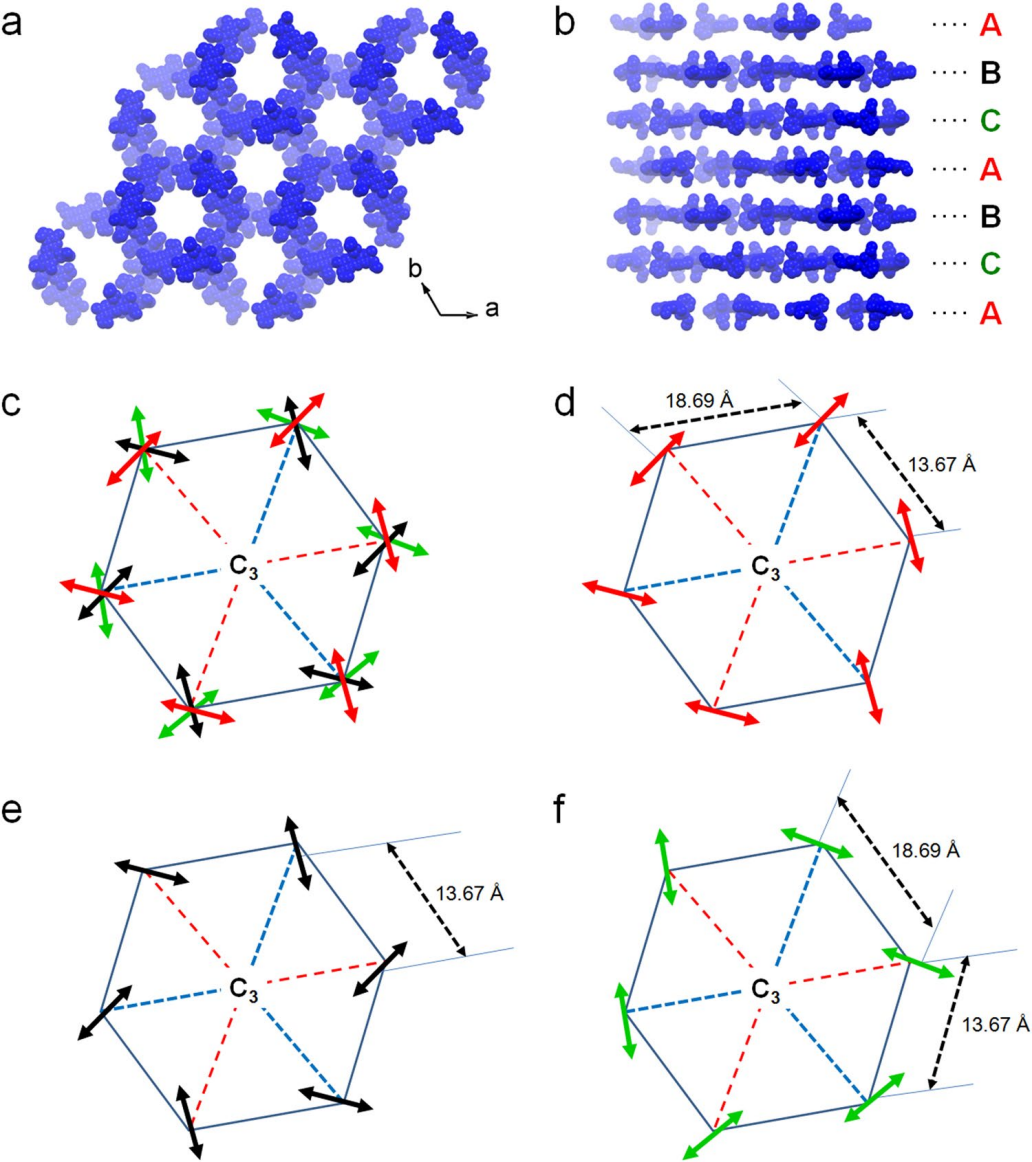
Spatial distribution of Rh6G and simplified symmetry of Rh6G. (**a**) Spatial distribution of Rh6G in Rh6G@In-BTB shown along the *c*-axis. (**b**) The corresponding view shown along the *a*-axis. In-BTB frameworks and hydrogen atoms are omitted for clarity. (**c**) The arrows represent the Rh6G dipoles, and different colors show the Rh6G cations in the neighboring hexagonal pores of the framework. (**d**) Red arrows indicate the first “A” layer of Rh6G. (**e**) Black arrows indicate the second “B” layer of Rh6G. (**f**) Green arrows indicate the third “C” layer of Rh6G. Intermolecular center-to-center distances of the Rh6G cations are indicated.

In our system, Rh6G@In-BTB showed a very uniform spatial distribution of Rh6G with unprecedented regularity inside the mesoscale channels of In-BTB, as shown in Fig. 5. Rh6G cations were encapsulated inside the pores of the In-BTB framework, and each Rh6G cation interacted with the In-BTB framework by H-bonding (Fig. 3). Notably, no π-π stacked dimers of Rh6G were observed. The Rh6G dimer formed through effective π-π stacking interaction has long been considered the main cause of the low quantum efficiency of emission due to either reabsorption of emission or self-quenching^36^. The Rh6G dipoles are represented as arrows to display how Rh6G is placed inside the In-BTB framework, as shown in Fig. 5c–f. The Rh6G cations are placed far enough from each other to prevent molecular aggregation in the solid state: the distances between the Rh6G monomers are 13.67 and 18.69 Å, which provide difficult forming π-π interactions for aggregation. The interlayer distance between the layers defined by the Rh6G cations is 13.9 Å (Fig. S9).

**Figure 6:**
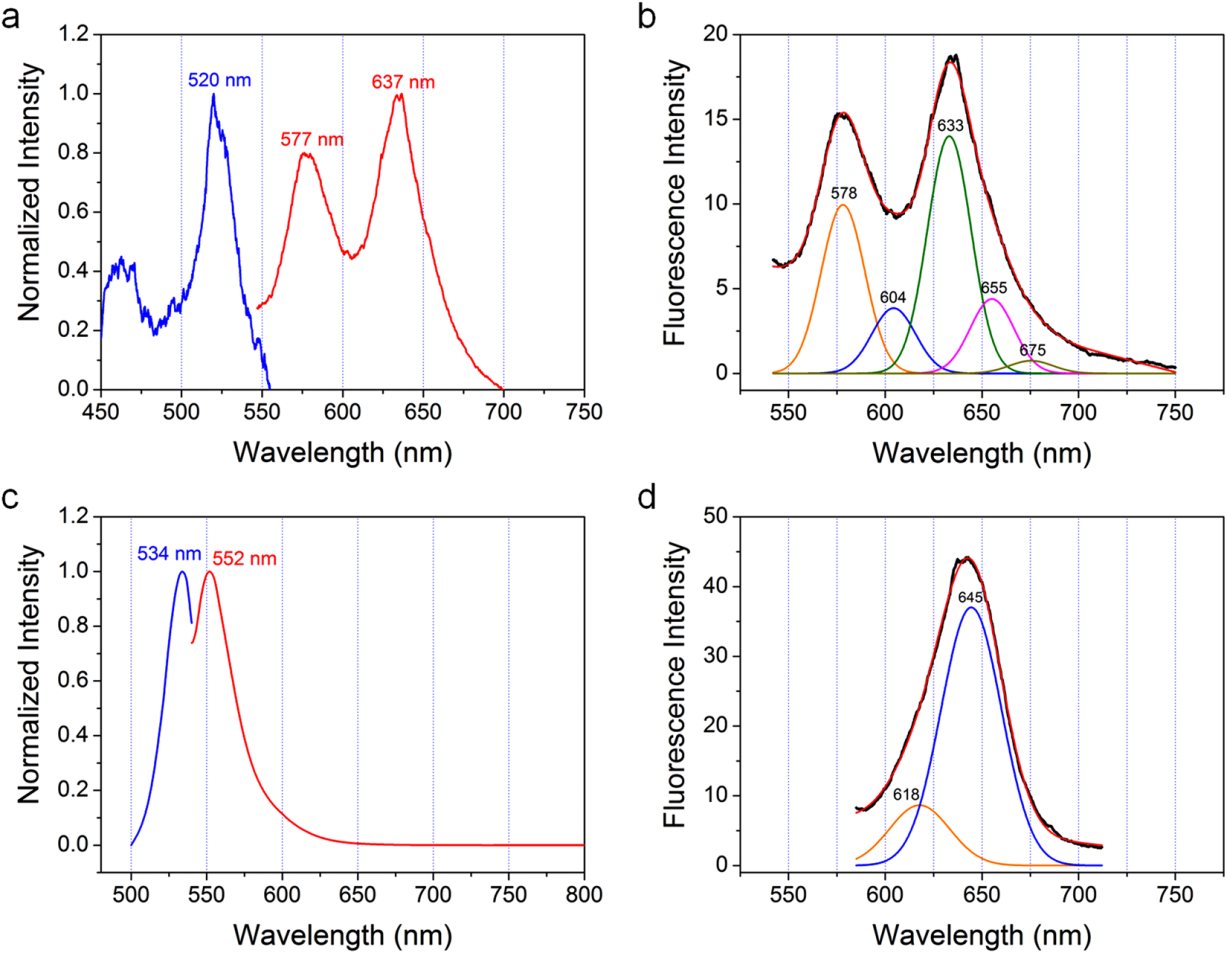
Emission spectra. (**a**) Normalized excitation (blue curve, λ_em_ = 632 nm) and emission (red curve, λ_ex_ = 520 nm) spectra of Rh6G@In-BTB. (**b**) Gaussian deconvoluted solid-state emission spectrum of Rh6G@In-BTB (λ_ex_ = 520 nm, *R*^2^ = 0.999, PeakFit v4.12). (**c**) Normalized excitation (blue curve, λ_em_ = 552 nm) and emission (red curve, λ_ex_ = 534 nm) spectra of a 1 × 10^−6^ M ethanolic solution of Rh6G. (**d**) Gaussian deconvoluted solid-state emission spectrum of bulk crystalline Rh6G measured at room temperature (λ_ex_ = 520 nm, *R*^2^ = 0.999, PeakFit v4.12).

The PL properties of Rh6G@In-BTB were investigated using solid-state fluorescence measurements. The crystalline sample was used without grinding to preserve the original state of Rh6G in In-BTB crystals, whose structure was obtained from X-ray crystallography. The solid-state excitation and fluorescence emission spectra of Rh6G@In-BTB are depicted in Fig. 6a. Surprisingly, the fluorescence spectrum of Rh6G@In-BTB is quite distinct from that of Rh6G dissolved in ethanol (1 × 10^−6^ M), as shown in Fig. 6c and Fig. S10. A single emission band was found at 552 nm (λ_ex_ = 534 nm) for Rh6G in ethanol. In contrast, the spectrum for Rh6G@In-BTB (λ_ex_ = 520 nm) showed two bands centered at 577 and 637 nm at room temperature, which can be further deconvoluted into five well-defined emission bands centered at 578, 604, 633, 655, and 675 nm, as depicted in Fig. 6b. The two emission maxima at 570 and 611 nm for Rh6G were previously observed in a frozen 2-propanol solution of Rh6G with a high concentration of 2 × 10^−2^ M at 77 K^37^. The former and latter emission bands are attributed to monomer emission and aggregate emission, respectively. The relative intensity ratio of I_Monomer_/I_Aggregate_ was reported to be 1:0.9. The ratio varied upon changing the measurement temperature. When the temperature was increased to 100 K, the ratio became 0.67:1. Further increasing the temperature led to the two bands merging at 140 K. At 184 K (the melting point of 2-propanol), only a single emission band at 563 nm corresponding to monomer emission was observed. This phenomenon was mainly attributed to the fact that the energy transfer from monomeric Rh6G to the aggregates became less efficient in solution despite the high concentration.

Zhu and coworkers also reported Rh6G@JUC-48, where JUC-48 is [Cd_3_(bpdc)_3_(DMF)]·5DMF·18H_2_O (bpdc = 4,4′-biphenyldicarboxylate), with large hexagonally shaped 1D channels with a dimension of 27.9 × 24.5 Å^38^. Unlike Rh6G@In-BTB, the emission spectrum of Rh6G@JUC-48 only showed a broad band at 563 nm (λ_ex_ = 541 nm) at room temperature. Decreasing the temperature to 77 K for Rh6G@JUC-48 only resulted in a slightly redshifted emission at 570 nm. No other redshifted emission bands with emission maxima greater than 600 nm were observed for Rh6G@JUC-48. Although it is difficult to analyze the positions of the Rh6G cations in Rh6G@JUC-48 due to a lack of crystallographic information, the single emission band can be conclusively assigned to diluted monomeric Rh6G distributed in the JUC-48 channels. Therefore, the four well-defined emission bands at 604, 633, 655, and 675 nm for Rh6G@In-BTB at room temperature can be attributed to new emission modes due to the unprecedented spatial ordering of the Rh6G cations in the pre-organized mesoscale channels of In-BTB. It is notable that these emissions are fundamentally different from the commonly observed aggregate emission in both the solution phase and solid state because Rh6G in Rh6G@In-BTB cannot form such aggregates. Moreover, the redshifts for the new emissions are prominent for Rh6G@In-BTB, owing to either efficient reabsorption of fluorescence by Rh6G in the channels and/or new energy transfer pathways. The control emission spectrum of bulk crystalline Rh6G (Sigma-Aldrich) excited at the same wavelength of 520 nm was also obtained for comparison. Bulk Rh6G only showed a broad single band deconvoluted into two emission bands at 618 and 645 nm, as shown in Fig. 6d. Notably, no monomer emission band was observed because of possible formation of aggregates through π-π stacking interactions, which usually induces a redshifted emission^34,35,39^. Therefore, the emission properties of Rh6G@In-BTB are quite different from those of bulk crystalline Rh6G.

Fluorescent guest molecules captured inside MOF channels often exhibited emission due to the efficient energy transfer from the excited framework to guest molecules when the framework is emissive^20^. In this case, the emission from the framework can excite the fluorescent guest molecules even if the excitation wavelength for the framework is not suitable to directly excite the guest molecules. The as-prepared In-BTB displayed an emission maximum at 369 nm when λ_ex_ was 333 nm (Fig. S11a). When Rh6G@In-BTB was excited at 333 nm, i.e., the excitation wavelength of as-prepared In-BTB, possible energy transfer from In-BTB to Rh6G occurred (Fig. S11b). Interestingly, two emission bands were also observed at 579 and 615 nm, together with the emission band at 382 nm. The latter emission band resulted from In-BTB. The former two bands obviously resulted from the captured Rh6G cations. For comparison, when the dilute solution of Rh6G in ethanol (1 × 10^−6^ M) was excited at 333 nm, a very weak single emission band at 552 nm was also observed (Fig. S12). These results may indicate that both energy transfer from In-BTB to Rh6G and direct excitation of Rh6G are possible for Rh6G@In-BTB upon excitation at 333 nm.

The encapsulating kinetics of Rh6G into In-BTB were monitored by UV/Vis absorption spectroscopy to estimate the amount of Rh6G in Rh6G@In-BTB (Fig. S13). Rh6G was gradually encapsulated into the channels of In-BTB until 150 h. The estimated encapsulation amount is 2.39 mmol per mmol of In-BTB. The Rh6G@In-BTB crystals were also digested in the mixture of DCl and DMSO-*d*_6_ (3:7 v/v%) for additional ^1^H-NMR spectroscopy measurements. The proton resonance signals from the digested BTB linkers and Rh6G did not overlap, and therefore, the simple integration of each resonance signal directly gave the amount of Rh6G encapsulated in Rh6G@In-BTB, i.e., 2.50 mmol Rh6G per mmol of In-BTB (0.336 mg/mg-In-BTB, Fig. S14a). This value is marginally different from the one measured from the encapsulation kinetics. The proton resonance signals from Rh6G were assigned based on the literature data^40^. Other dye@In-BTBs were also digested in DCl and DMSO-*d*_6_ (3:7 v/v%) for ^1^H-NMR spectroscopy measurements (Table S2). NBA from NBA@In-BTB and DEOCy from DEOCy@In-BTB did not give interpretable resonance signals possibly because of decomposition in acidic solution. The ^1^H-NMR spectra of digested AOG@In-BTB, CV@In-BTB and DMMP@In-BTB are shown in Figs S14b and S15, respectively. The amounts of captured AOG, CV, and DMMP are 1.08 mmol AOG (0.106 mg/mg-In-BTB), 2.50 mmol CV (0.286 mg/mg-In-BTB), and 2.86 mmol DMMP (0.293 mg/mg-In-BTB) per mmol of In-BTB, respectively.

### Quantum efficiency

Green and Buckely also attempted to encapsulate Rh6G in a poly(methyl methacrylate) (PMMA) host to avoid uncontrolled aggregation of Rh6G and to investigate the solid-state concentration quenching mechanism of Rh6G^41^. The concentration of encapsulated Rh6G was carefully controlled to fine-tune the distances between the Rh6G cations and subsequently measure the absolute QYs using an integrating sphere. It was revealed that if the distance between the Rh6G cations was less than 3.5 nm, the QY was very low (<0.1) due to enhanced non-radiative de-excitation pathways. However, when the distance was approximately 12 nm, high QYs were observed in the range of 0.8 to 0.9. Despite these encouraging results, it was concluded that there was no way to measure the aggregation of Rh6G. In contrast, our system has full positional information of the Rh6G cations in the host. To estimate the quantum efficiency of Rh6G@In-BTB, the QY was measured by an absolute method using an integrating sphere. The QY of 0.25 for Rh6G@In-BTB is more than 3-fold higher than the value of 0.07 for bulk crystalline Rh6G. Despite the closest contact distance of the Rh6G cations in Rh6G@In-BTB being much less than 3.5 nm, Rh6G@In-BTB exhibited a QY higher than 0.1 possibly due to the ideal positioning in the MOF channels without forming aggregates.

### Encapsulated NBA system

The encapsulation amount of NBA is 3.11 mmol per mmol of In-BTB (0.319 mg/mg-In-BTB, Fig. S13 and Table S2). Two NBA cations formed a dimer through effective π-π stacking in NBA@In-BTB, as shown in Fig. 3b. The effective formation of dimers may be the result of the small molecular dimension of NBA compared to Rh6G. It is worthwhile to mention that the NBA dimer is exclusively formed in the channels of In-BTB. The solid-state emission spectrum of NBA@In-BTB was investigated (Fig. S16a). Only a single emission band at 642 nm with a very weak intensity was observed. The effective formation of a dimer between two NBA ions inside the mesoscale channels is responsible for the weak emission band due to self-quenching of the fluorescence^42^. In contrast, the emission spectrum of NBA in ethanol (1 × 10^−6^ M) showed an emission band centered at 598 nm (λ_ex_ = 500 nm) (Fig. S16b). This exemplary case also shows that detailed crystallographic information of guest dyes in MOF channels can be very informative to interpret their corresponding PL properties.

### Encapsulated AOG system

The excitation and emission spectra for AOG@In-BTB were investigated (Fig. S17). A single emission band at 559 nm was observed at λ_ex_ = 537 nm. The Z-stack CLSM images of AOG@In-BTB were also obtained (Fig. S18). A very uniform orange-colored emission from the AOG cations was observed for two different crystals. Both crystals showed very similar CLSM images to Rh6G@In-BTB. The penetration depth of the AOG cations is approximately 6 μm (Fig. S19).

### Fluorescence lifetime measurements

**Figure 7:**
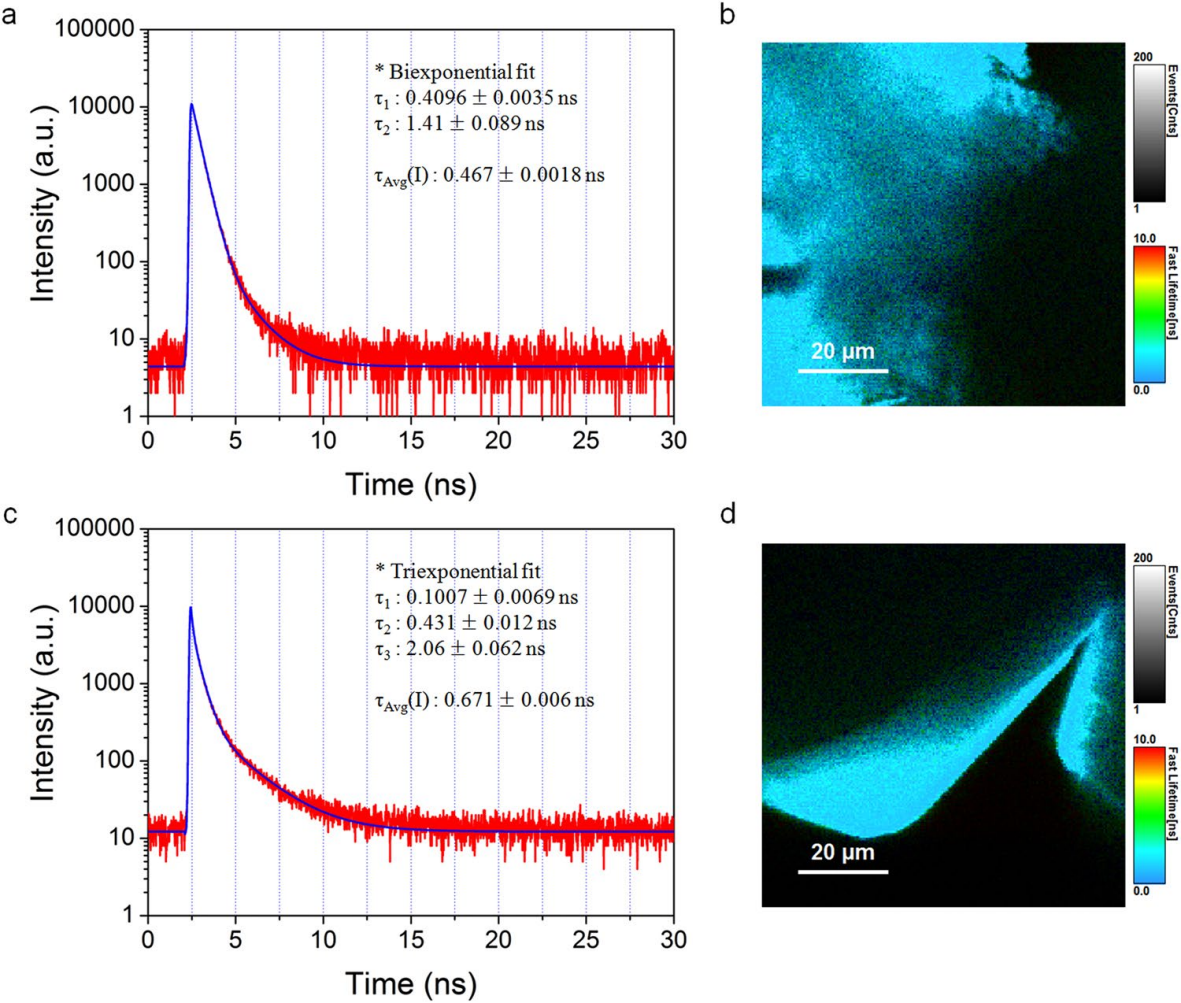
Fluorescence lifetime measurements. (**a**) Fluorescence decay curve for bulk crystalline Rh6G (λ_ex_ = 470 nm). (**b**) Fluorescence lifetime imaging (FLIM) image of the bulk crystalline Rh6G. (**c**) Fluorescence decay curve for Rh6G@In-BTB (λ_ex_ = 470 nm). (**d**) FLIM image of Rh6G@In-BTB.

The photophysical properties of dyes captured in MOF channels can be altered due to their different chemical environment^18^. The fluorescence decay profile of the captured dye as well as fluorescence properties can be changed. Thus, the fluorescence lifetimes of the representative samples Rh6G@In-BTB and bulk crystalline Rh6G were investigated by time-resolved PL (TRPL) measurements using a time-correlated single-photon counting (TCSPC) technique. The fluorescence decay curves are shown in Fig. 7. The decay curve for bulk crystalline Rh6G was fitted biexponentially, and the lifetimes are τ_1_ = 0.4096 ± 0.0035 and τ_2_ = 1.41 ± 0.089 ns whereas the curve for Rh6G@In-BTB was fitted triexponentially, and the lifetimes are τ_1_ = 0.1007 ± 0.0069, τ_2_ = 0.431 ± 0.012, and τ_3_ = 2.06 ± 0.062 ns. The Rh6G cations in Rh6G@In-BTB exhibited an increased intensity average lifetime (τ_Avg_(I) = 0.671 ± 0.006 ns) compared to bulk crystalline Rh6G (τ_Avg_(I) = 0.467 ± 0.0018 ns), suggesting that the captured Rh6G cations tend to maintain longer excited states than bulk Rh6G^43^.

In summary, to elucidate the crystallographic information of the various dyes encapsulated in the anionic In-BTB framework (dye@In-BTBs) and the PL properties of the encapsulated dyes, we chose six different cationic dyes for encapsulation. The cationic dyes include rhodamine 6G (Rh6G), nile blue A (NBA), Astrazon Orange G (AOG), crystal violet (CV), 3,3′-diethyloxacarbocyaine iodide (DEOCy), and 2-[4-dimethylamino]styryl)−1-methylpyridinium (DMMP). The dyes are thought to be encapsulated through simple cation-exchange processes. The crystal structures of dye@In-BTBs were completely solved, and the PL spectra of three of these species were investigated. The successful refinements of the crystal structures of dye@In-BTBs can be attributed to the ideal dimensions of the cationic dyes and mesoscale channels of the anionic framework of In-BTB. Particularly, Rh6G@In-BTB exhibited distinct PL properties due to the unprecedented spatial ordering and isolation of the Rh6G cations in the mesoscale channels compared to free Rh6G in ethanol and bulk crystalline Rh6G. Both monomer emission and new emissions at longer wavelengths were identified at room temperature. The corresponding QY was higher than that of the control bulk crystalline Rh6G. NBA cations captured as a dimeric form did not give strong emission due to self-quenching. These results suggest that the photophysical properties of dyes can be tuned in a controllable manner by simply encapsulating into suitable channels of MOFs. There are numerous high surface MOFs having different charges in the frameworks^44–46^. Thus, we envision that various MOF crystals with encapsulated organic or inorganic dyes can be very useful for diverse sophisticated optical applications.

## Exprimental Methods

### Materials

1,3,5-Benzenetribenzoic acid (H_3_BTB) was prepared according to previous literature^47^. In(NO_3_)_3_ hydrate, rhodamine 6G (Rh6G), nile blue A (NBA), Astrazon Orange G (AOG), crystal violet (CV), 3,3′-diethyloxacarbocyaine iodide (DEOCy), 2-[4-dimethylamino]styryl)-1-methylpyridinium (DMMP), deuterium chloride solution (35 wt% in D_2_O), and DMSO-*d*_6_ were purchased from Sigma-Aldrich and used as received. *N*,*N*-Diethylformamide (DEF) was purchased from TCI and used without further purification. All other reagent grade solvents were used as received.

### Preparation of [(CH_3_CH_2_)_2_NH_2_]_3_[In_3_(C_27_H_15_O_6_)_4_]∙10(C_5_H_11_NO)·14(H_2_O) (In-BTB)

In-BTB was prepared based on published literature^22^.

### Encapsulation of Rh6G

The as-prepared In-BTB (10 mg) was added to a 2.0 mM solution of Rh6G dissolved in ethanol (10 mL) in a vial and gently shaken at room temperature. The vial was covered with aluminum foil. Aliquots of the solution were periodically withdrawn and diluted with ethanol to give a 250-fold diluted solution for quantification by UV-Vis spectroscopy. The measured molar extinction coefficient of 1.0 mM Rh6G in ethanol was 106463 M^−1^ cm^−1^ at λ_max_ = 531.6 nm. The X-ray crystallographic data of Rh6G@In-BTB were collected directly using the retrieved crystals from the mixture after 7 d.

### Encapsulation of NBA

The mixture containing as-prepared In-BTB (10 mg) and a 2.0 mM ethanol solution of NBA (10 mL) was gently shaken at room temperature in dark condition. Aliquots of the solution were periodically withdrawn and 200-fold diluted with ethanol for quantification by UV-Vis spectroscopy. The measured molar extinction coefficient of 1.0 mM NBA in ethanol was 72769 M^−1^ cm^−1^ at λ_max_ = 629.0 nm. The crystallographic data of NBA@In-BTB were collected using the retrieved crystals from the mixture after 7 d.

### Encapsulation of AOG

The as-prepared In-BTB (10 mg) was added to a saturated ethanol solution of AOG (10 mL) and gently shaken at room temperature in dark condition. The crystallographic data of AOG@In-BTB were collected using the retrieved crystals from the mixture after 7 d.

### Encapsulation of CV

The as-prepared In-BTB (10 mg) was added to a saturated ethanol solution of CV (10 mL) and gently shaken at room temperature in dark condition. The crystallographic data of CV@In-BTB were collected using the retrieved crystals from the mixture after 7 d.

### Encapsulation of DEOCy

The as-prepared In-BTB (10 mg) was added to a 2.0 mM ethanol solution of DEOCy (10 mL) and gently shaken at room temperature in dark condition. Aliquots of the solution were periodically withdrawn and 300-fold diluted with ethanol for quantification by UV-Vis spectroscopy. The measured molar extinction coefficient of 2.0 mM NBA in ethanol was 145242 M^−1^ cm^−1^ at λ_max_ = 481.1 nm. The crystallographic data of DEOCy@In-BTB were collected using the retrieved crystals from the mixture after 7 d.

### Encapsulation of DMMP

The as-prepared In-BTB (10 mg) was added to a 2.0 mM ethanol solution of DMMP (10 mL) and gently shaken at room temperature in dark condition. Aliquots of the solution were periodically withdrawn and 100-fold diluted with ethanol for quantification by UV-Vis spectroscopy. The measured molar extinction coefficient of 2.0 mM DMMP in ethanol was 42947 M^−1^ cm^−1^ at λ_max_ = 465.2 nm. The crystallographic data of DMMP@In-BTB were collected using the retrieved crystals from the mixture after 7 d.

### X-ray crystallography

The X-ray intensity data of Rh6G@In-BTB were measured with a Bruker AXS X8 Prospector system equipped with a multilayer monochromator and a Cu IμSTM microfocus sealed-tube X-ray source (λ* = *1.54178 Å). The X-ray diffraction data for other dye@In-BTB MOFs were collected by a Bruker APEX-II diffractometer equipped with a monochromator and a Mo Kα X-ray source (λ = 0.71073 Å). Rh6G@In-BTB and NBA@In-BTB crystals were mounted on the loop for X-ray data collections at 223 K. The other dye@In-BTB crystals were inserted into a Lindemann capillary. The capillary was then filled with the mother liquor and sealed off by epoxy glue for data collection at room temperature. Bruker-SAINT software package was used for the integration and scaling of the collected CCD data. The crystal structures were solved and refined using SHELXL 2014^48^. All hydrogen atoms were placed in their calculated positions. SQUEEZE/PLATON function was employed in structural refinement. The crystallographic data are listed in Table 1. Structural information was deposited at the Cambridge Crystallographic Data Center.

### Instrumentation

NMR spectra were recorded using a Bruker Ascend 400 (400.13 MHz for ^1^H) spectrometer. The proton chemical shifts of samples were calibrated with respect to the reference proton resonance signals occurring from the protic residues of the deuterated solvents. Elemental compositions of MOF samples were analyzed at the Organic Chemistry Research Center (Seoul, Korea). PXRD spectra were recorded on a Bruker D8 Focus diffractometer (40 kV, 30 mA, step size = 0.02°). SEM images were recorded on a Coxem CX-100S (accelerating voltage = 20 kV, Korea). UV/Vis spectra were collected by a Scinco S-3100 spectrophotometer. Diffuse reflectance UV/Vis spectra were collected by a Jasco V-550 spectrophotometer. FT-IR spectra were obtained with Jasco FT/IR-4100 spectrometer using the KBr pellet method. Solid-state fluorescence spectra were collected using a Hitachi F-7000 fluorescence spectrophotometer. Absolute quantum yields were measured using a Quanta-*φ* integrating sphere system connected to a Fluorolog-3 fluorescence spectrophotometer (Horiba).

### Photoluminescence lifetime measurements

Time-resolved photoluminescence (TRPL) imaging was performed using an inverted-type scanning confocal microscope (MicroTime-200, Picoquant, Germany) with a 60× (water) objective. The measurements were performed at the Korea Basic Science Institute (KBSI), Daegu Center. A single-mode pulsed diode laser (470 nm with ~30 ps pulse width and an average power of < 1 μW) was used as an excitation source. A dichroic mirror (490 DCXR, AHF), a long-pass filter (HQ500lp, AHF), and a single-photon avalanche diode (SPAD; PDM series, MPD) were used to collect the emission from each sample. A time-correlated single-photon counting (TCSPC) technique was used to count emitting photons. TRPL images with a dimension of 80 × 80 μm^2^, which consisted of 200 × 200 pixels, were recorded using a time-tagged time-resolved (TTTR) data acquisition route. The acquisition time was 2 ms for each pixel. Exponential fittings for the obtained PL decays were performed using the Symphotime-64 software.

## Electronic supplementary material

Supplementary Information
