# Supplementary material for: Photophysical properties of cationic dyes captured in the mesoscale channels of micron-sized metal-organic framework crystals
Source: Sci Rep. 2018 Jun 29;8:9838. doi: 10.1038/s41598-018-28080-y (PMC6026151; doi:10.1038/s41598-018-28080-y)
Supplement: Supplementary file 1 — Supplementary Information [file 41598_2018_28080_MOESM1_ESM.doc]

**Supplementary Information**

**Photophysical properties of cationic dyes captured in the mesoscale channels of micron-sized metal-organic framework crystals**

In-Hwan Choi^1^, Suk Bin Yoon^1^, Seong Huh^*,1^, Sung-Jin Kim^2^ and Youngmee Kim^*,2^

^1^Department of Chemistry and Protein Research Center for Bio-Industry, Hankuk University of Foreign Studies, Yongin 17035, Korea

^2^Institute of Nano-Bio Technology and Department of Chemistry and Nano Science, Ewha Womans University, Seoul 03760, Korea

Correspondence and requests for materials should be addressed to S.H. (email: shuh@hufs.ac.kr) or to Y.K. (email: ymeekim@ewha.ac.kr).


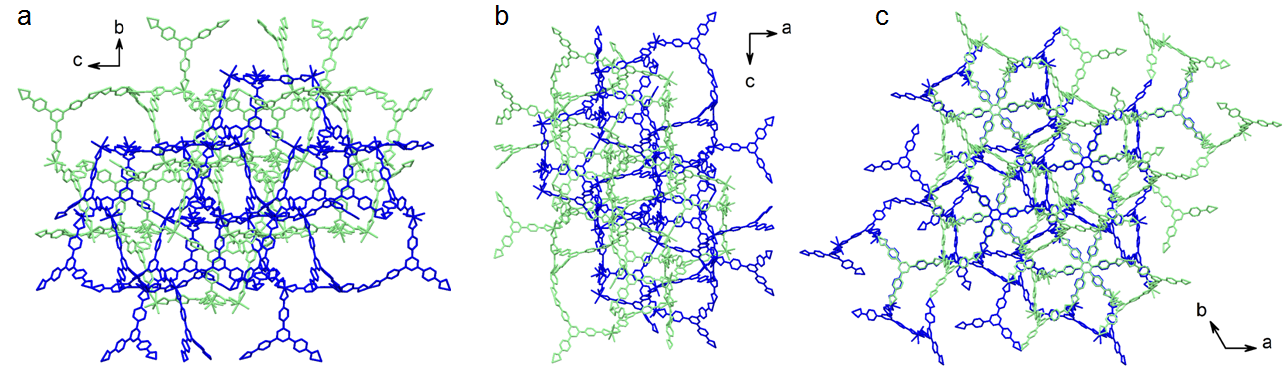


**Figure S1**. 2-Fold interpenetrated 3D framework structure of the as-prepared In-BTB along (a) *a*-, (b) *b*-, and (c) *c*-axes.


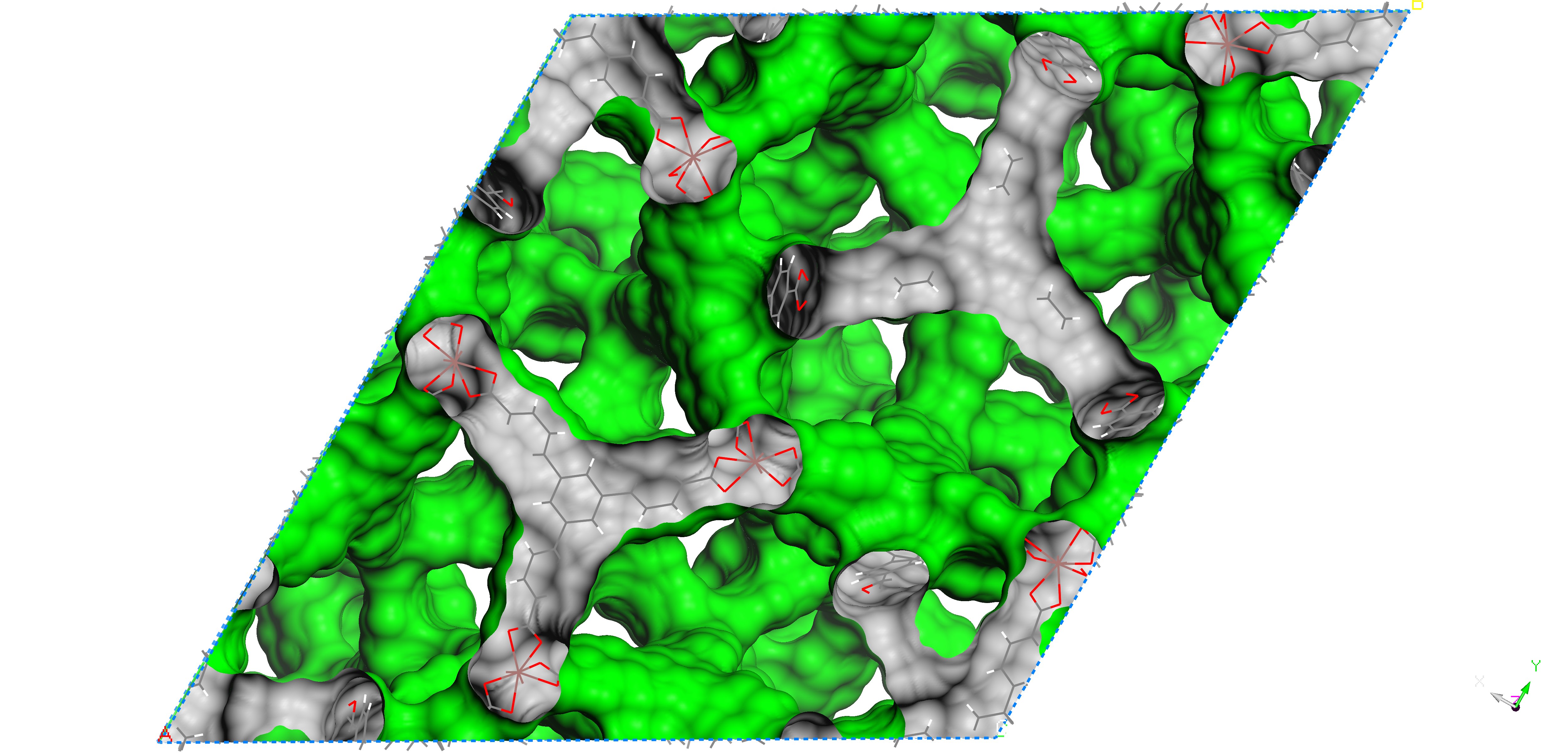


**Figure S2**. Connolly surface of cation- and solvent-free In-BTB viewed down the *c*-axis (1.4 Å probe radius). Grey and green colors indicate the exterior and interior surfaces, respectively (Materials Studio 4.4).


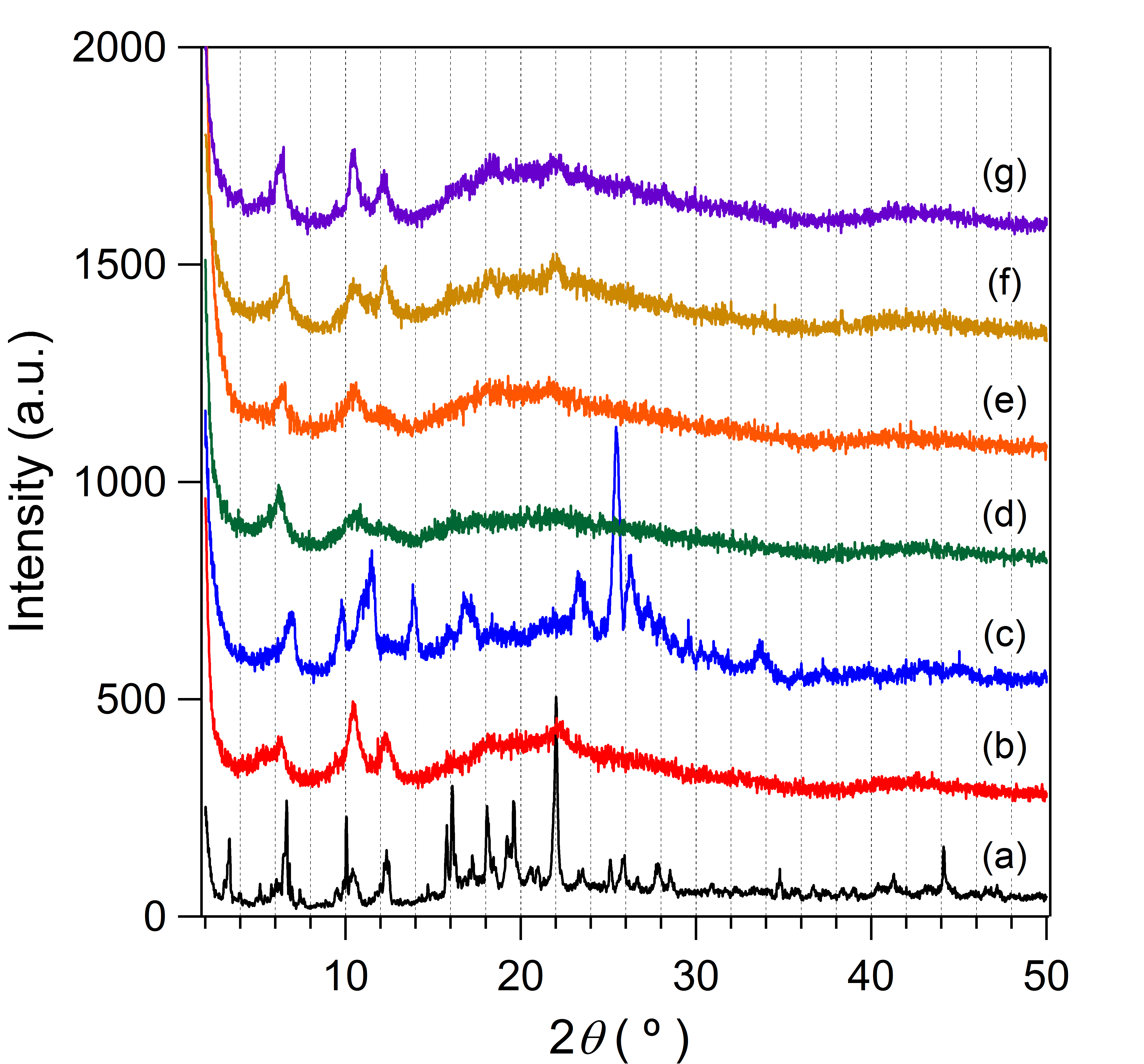


**Figure S3**. PXRD patterns of dye@In-BTBs. (a) As-prepared In-BTB, (b) Rh6G@In-BTB, (c) NBA@In-BTB, (d) AOG@In-BTB, (e) CV@In-BTB, (f) DEOCy@In-BTB, and (g) DMMP@In-BTB.


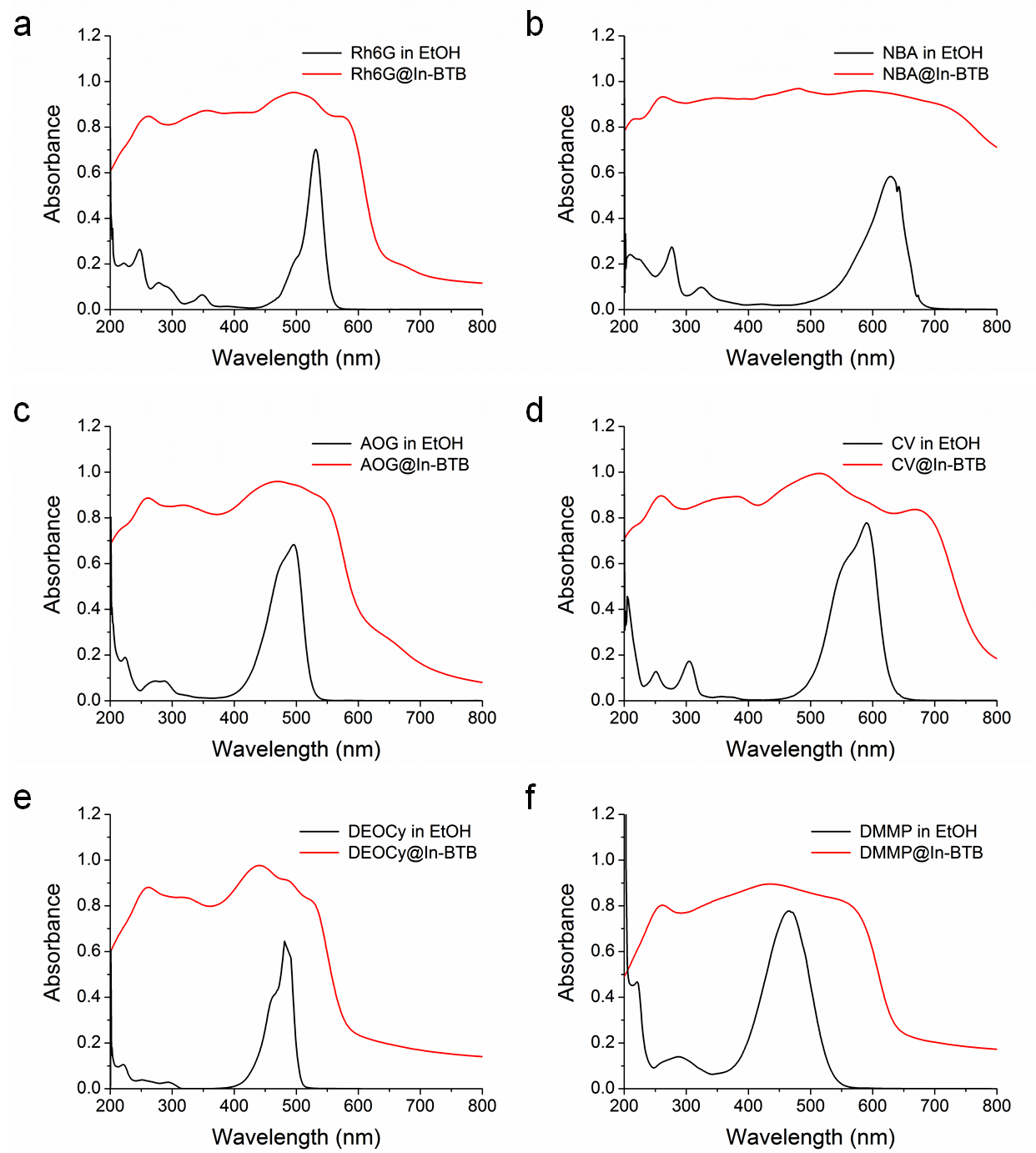


**Figure S4**. Solid-state diffuse reflectance UV/Vis spectra of dye@In-BTBs. Rh6G@In-BTB (a), NBA@In-BTB (b), AOG@In-BTB (c), CV@In-BTB (d), DEOCy@In-BTB (e), and DMMP@In-BTB (f). The absorption spectra of free dyes were measured in ethanol.


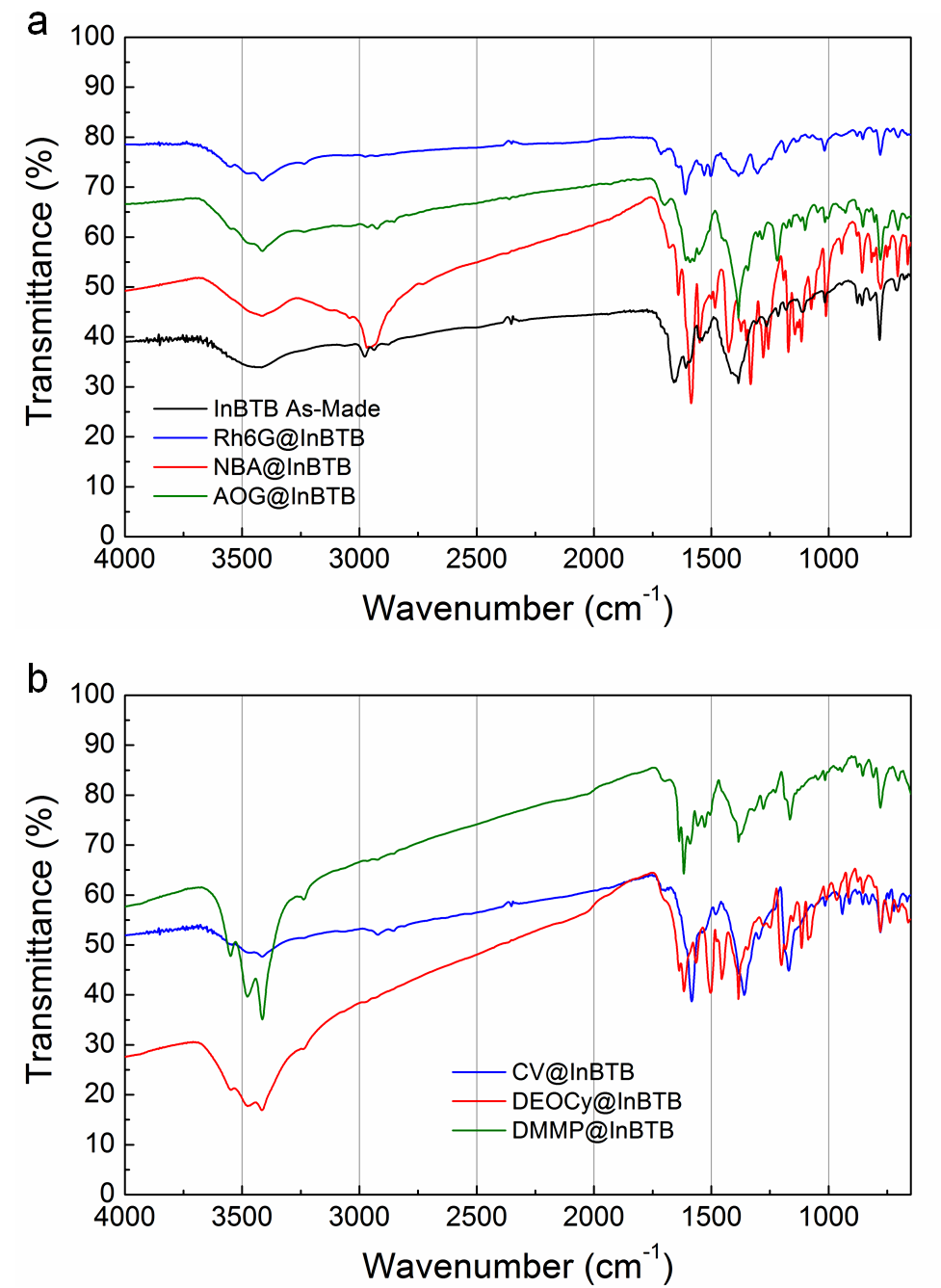


**Figure S5**. FT-IR spectra of dye@In-BTBs.


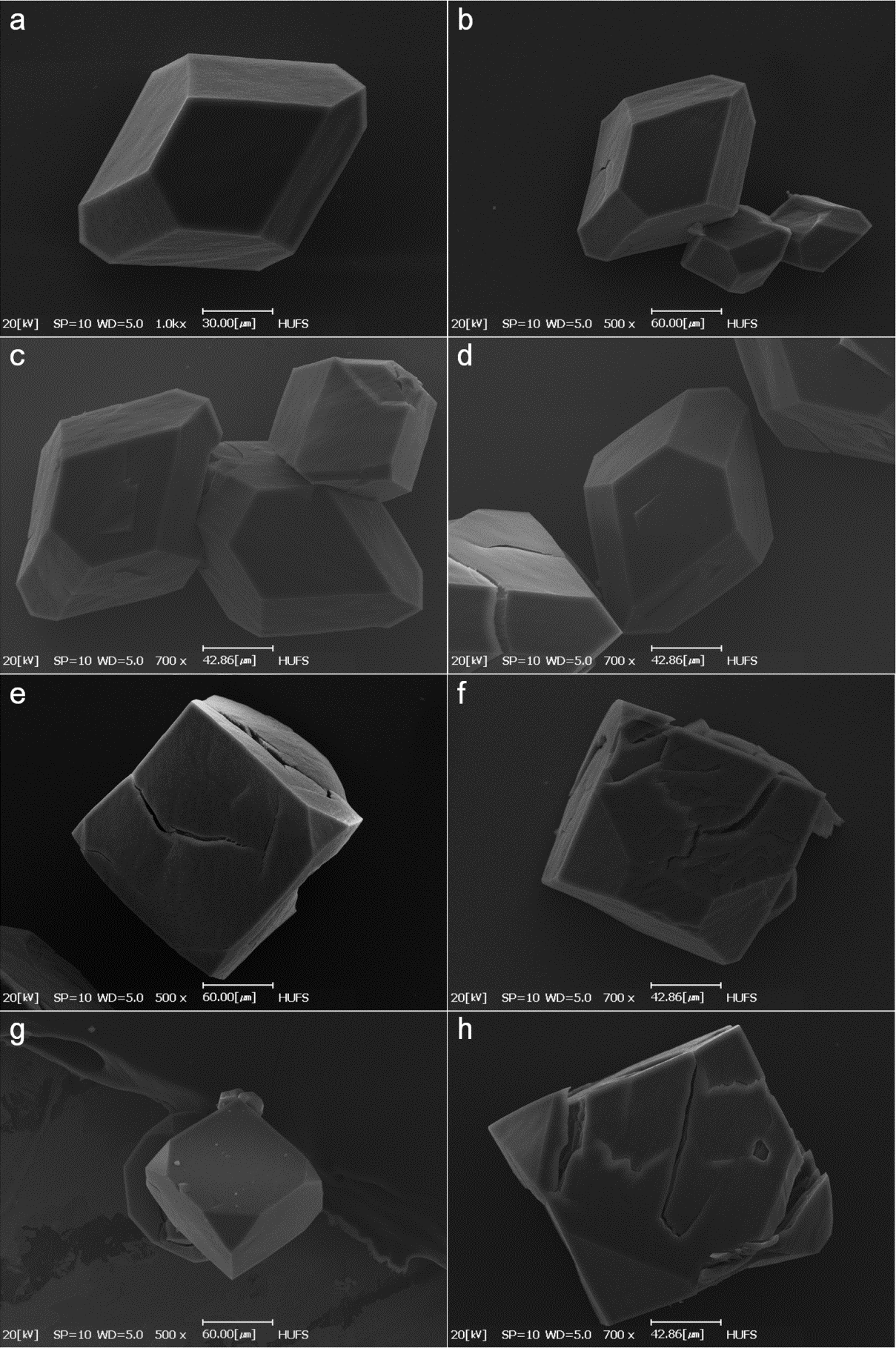


**Figure S6**. SEM images of as-prepared In-BTB. Two different crystal morphologies are shown: (a-d) crystals with pentagonal facets and (e-h) truncated cube.


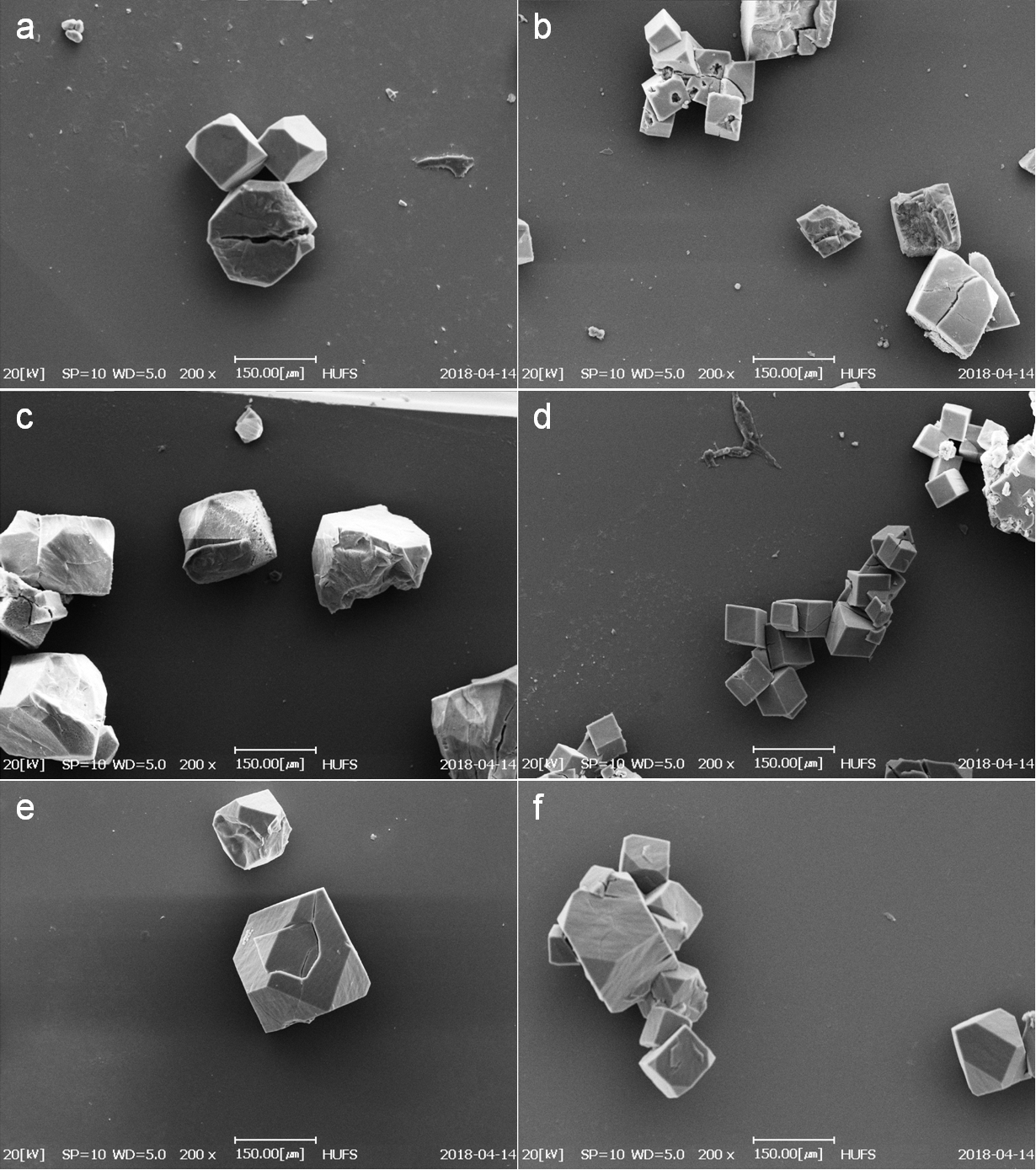


**Figure S7**. SEM images of dye@In-BTBs. (a) Rh6G@In-BTB, (b) NBA@In-BTB, (c) AOG@In-BTB, (d) CV@In-BTB, (e) DEOCy@In-BTB, and (f) DMMP@In-BTB.


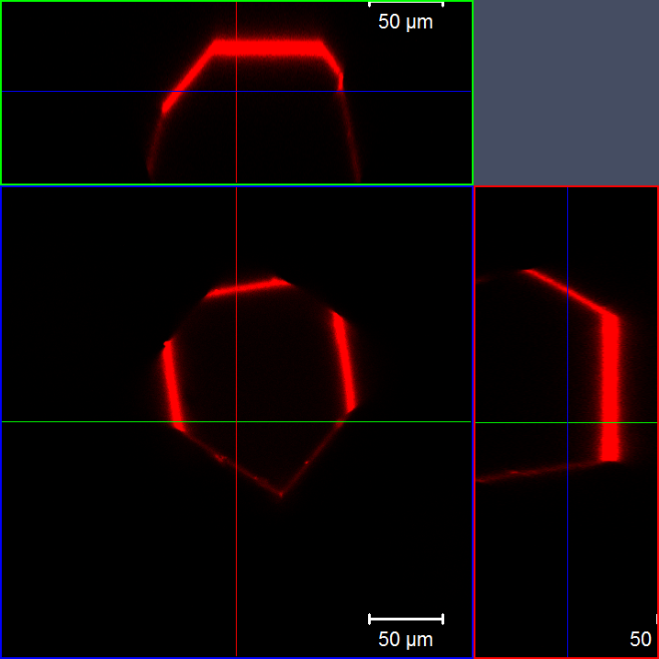


**Figure S8**. A sliced CLSM image of Rh6G@In-BTB (λ_ex_ = 488 nm, λ_em_ = 518 nm).


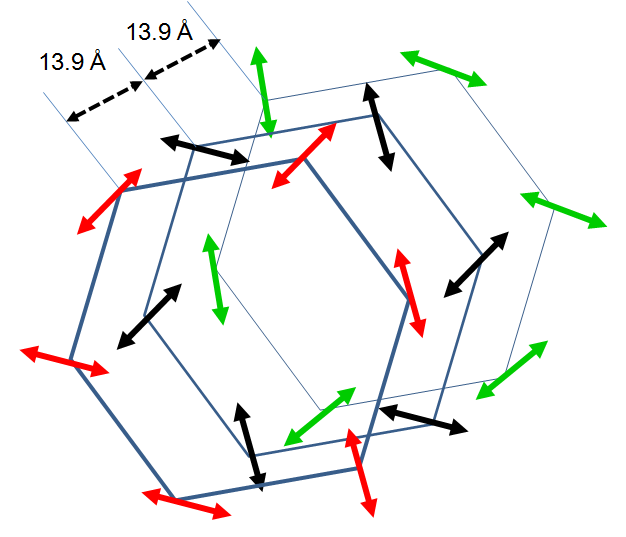


**Figure S9**. The interlayer distances between the layers defined by Rh6G cations. The view is slightly tilted from the *c*-axis. The arrows represent Rh6G dipoles, and different colors show Rh6G dipoles in different layers. The Rh6G layer is a three-sequence layer, ABCABC. In-BTB frameworks and hydrogen atoms are omitted for clarity.


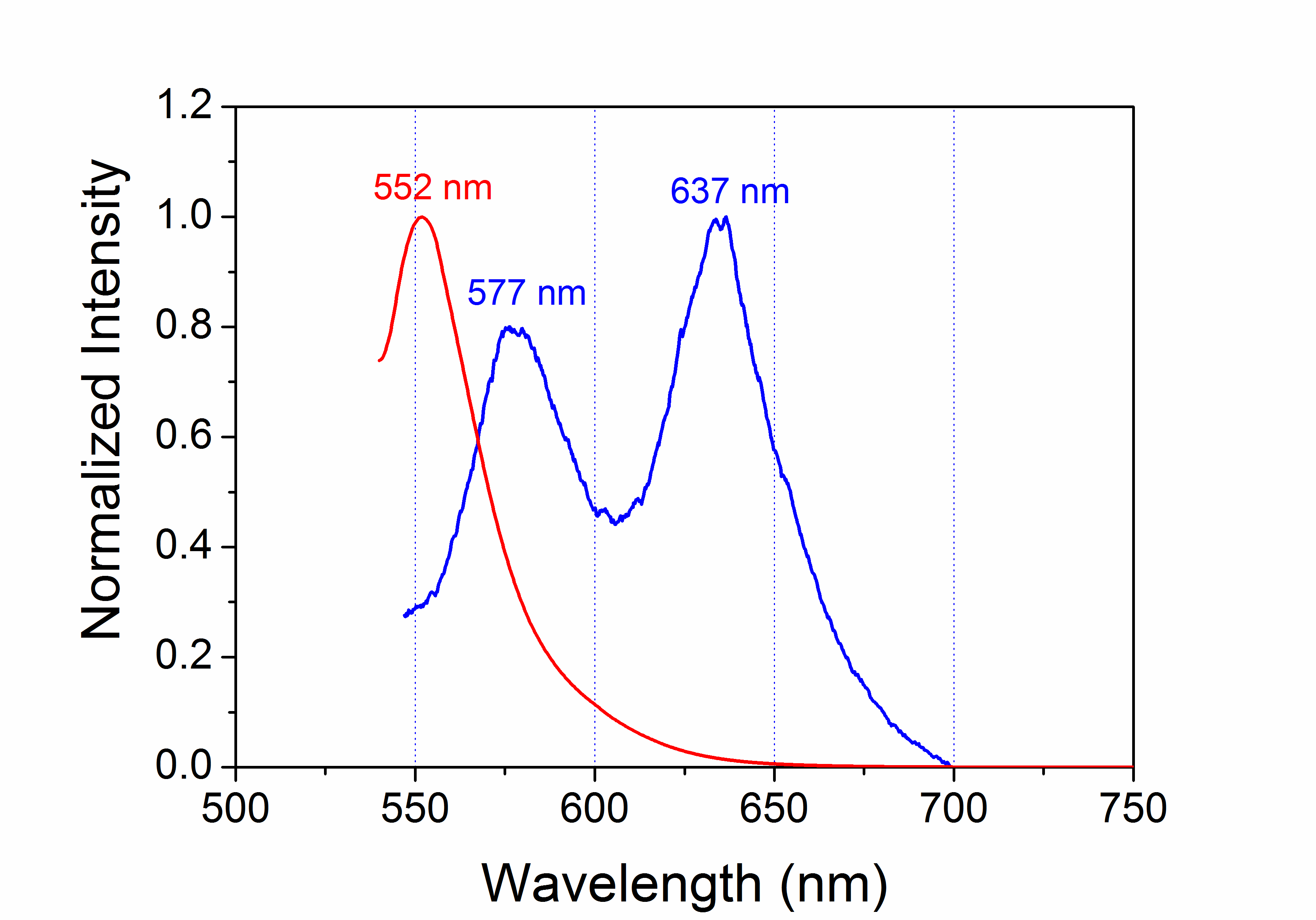


**Figure S10**. Comparison of normalized emission spectra of Rh6G@In-BTB (λ_ex_ = 520 nm, blue line) and 1 × 10^-6^ M ethanolic solution of Rh6G (λ_ex_ = 534 nm, red line).

**
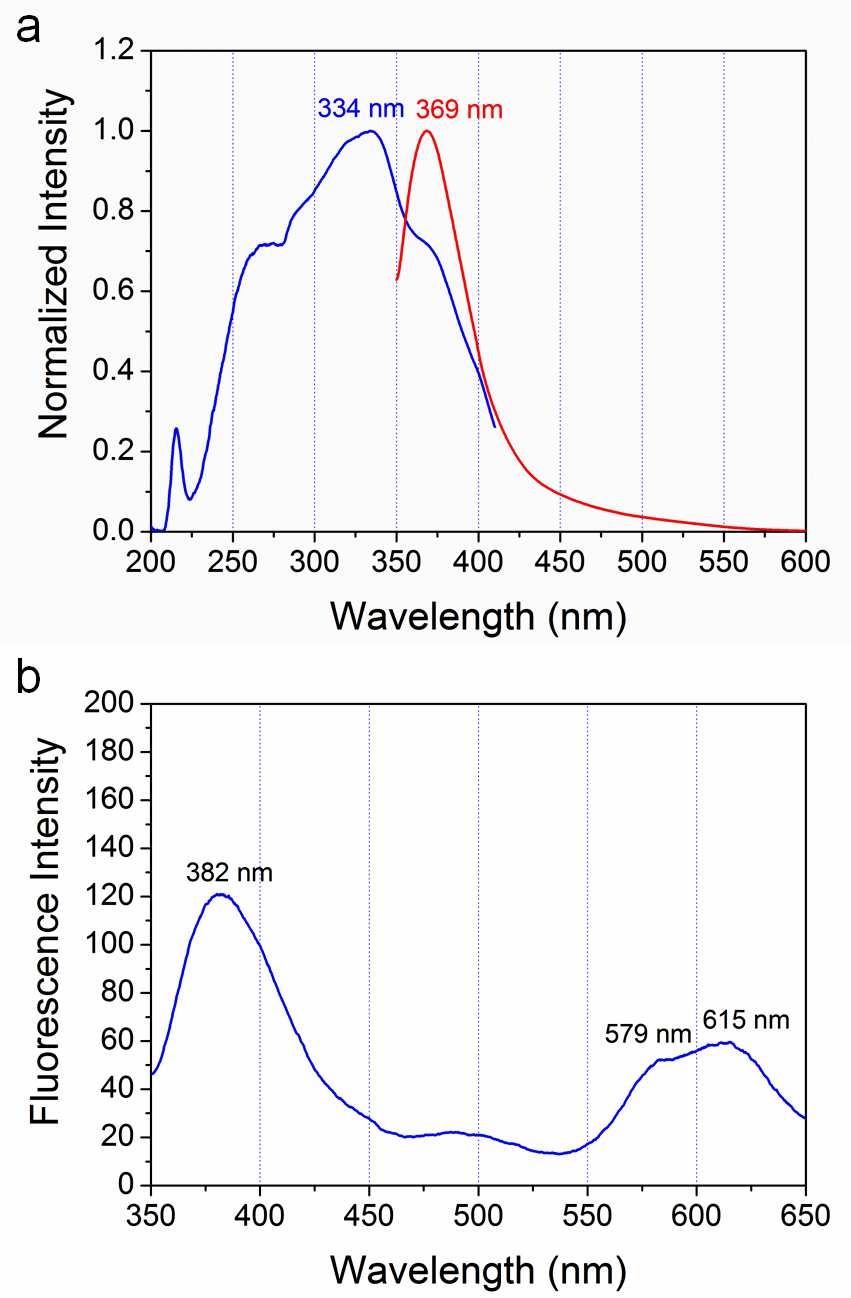
**

**Figure S11**. (a) Normalized excitation (blue, λ_em_ = 377 nm) and emission (red, λ_ex_ = 333 nm) spectra of as-prepared In-BTB. (b) Emission spectrum of Rh6G@In-BTB (λ_ex_ = 333 nm).

**
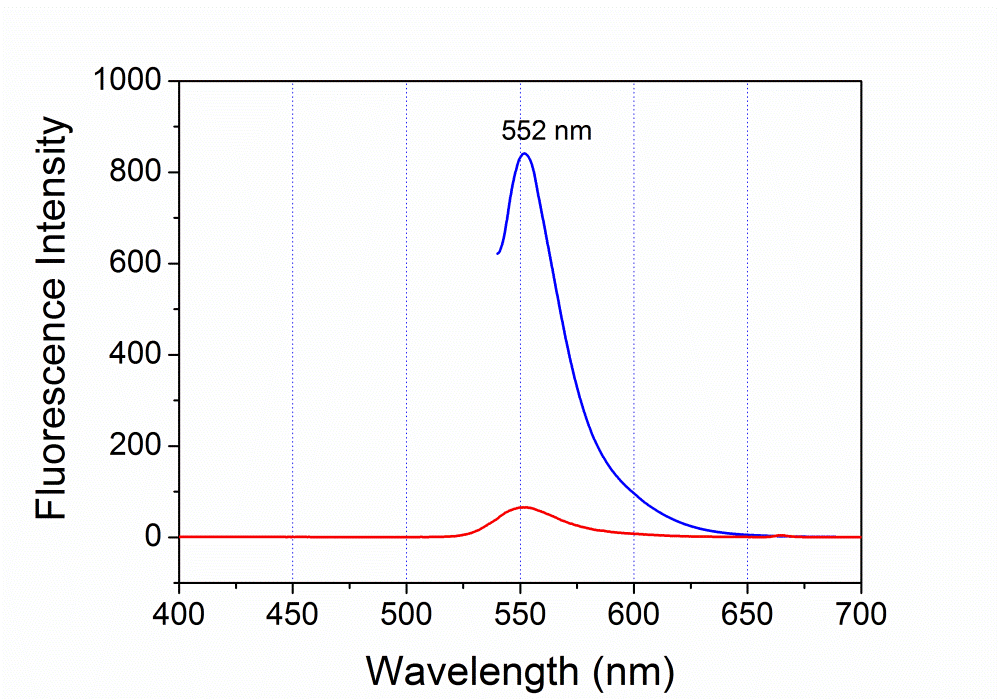
**

**Figure S12**. Comparison of emission spectra of 1 × 10^-6^ M ethanolic solution of Rh6G at different excitation wavelengths (λ_ex_ = 520 nm, blue line; λ_ex_ = 333 nm, red line).

**
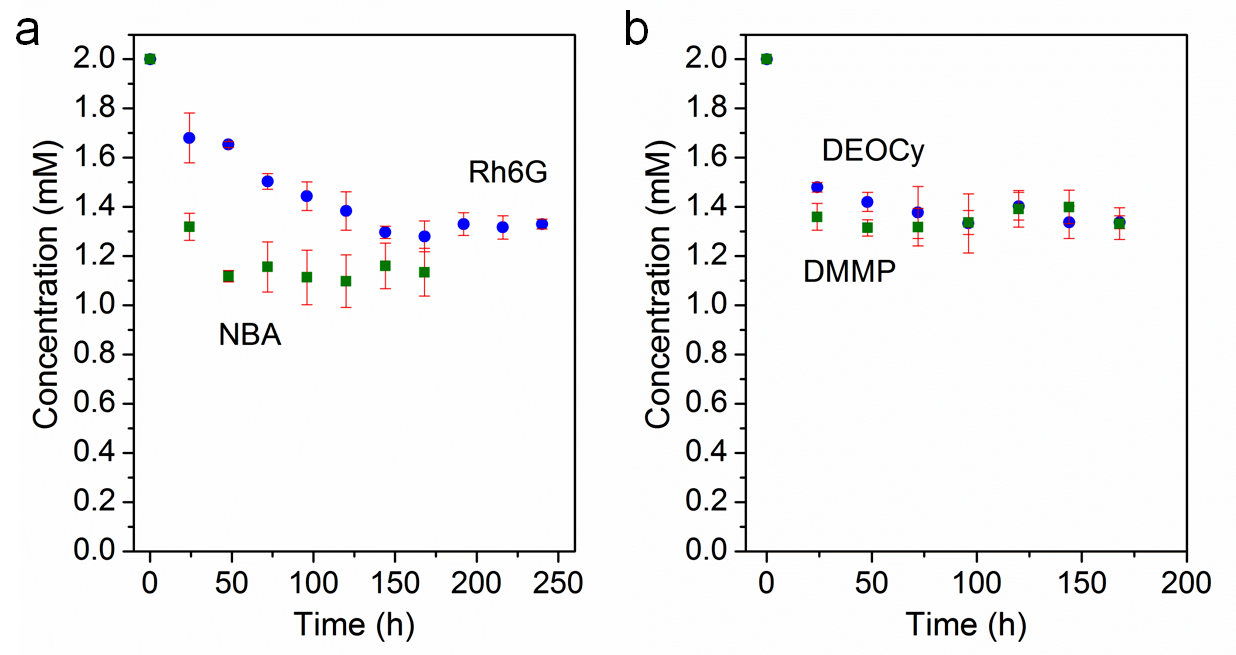
**

**Figure S13**. (a) Timecourses of the encapsulation of Rh6G and NBA by as-prepared In-BTB. (b) Timecourses of the encapsulation of DEOCy and DMMP by as-prepared In-BTB. The measurements were performed in triplicate.

**
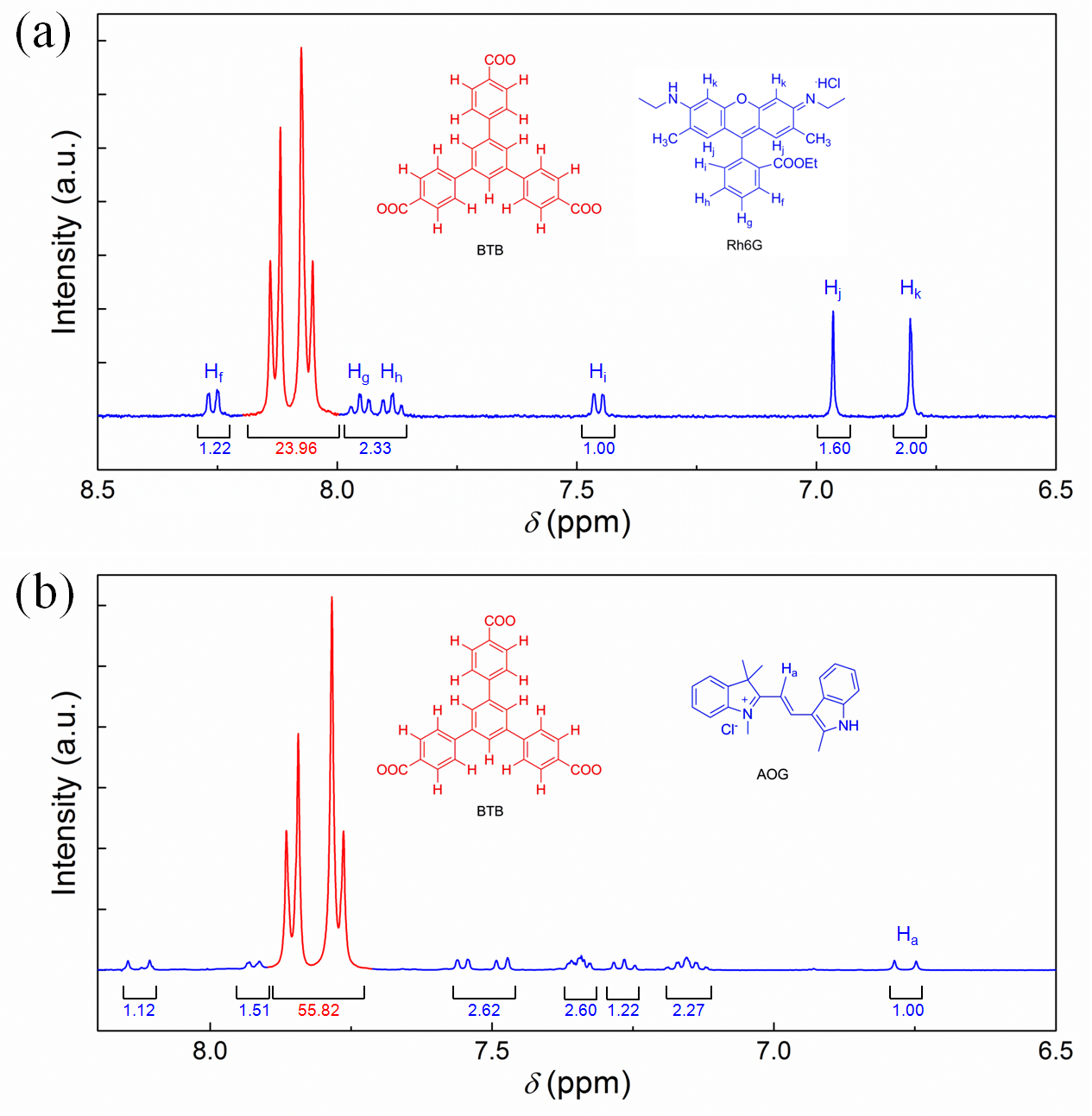
**

**Figure S14**. ^1^H-NMR spectra of the digested (a) Rh6G@In-BTB and (b) AOG@In-BTB in the mixture of DCl and DMSO-*d*_6_ (3:7 v/v%). The red signals indicate the resonance signals from BTB linker and the blue signals from Rh6G and AOG.

**
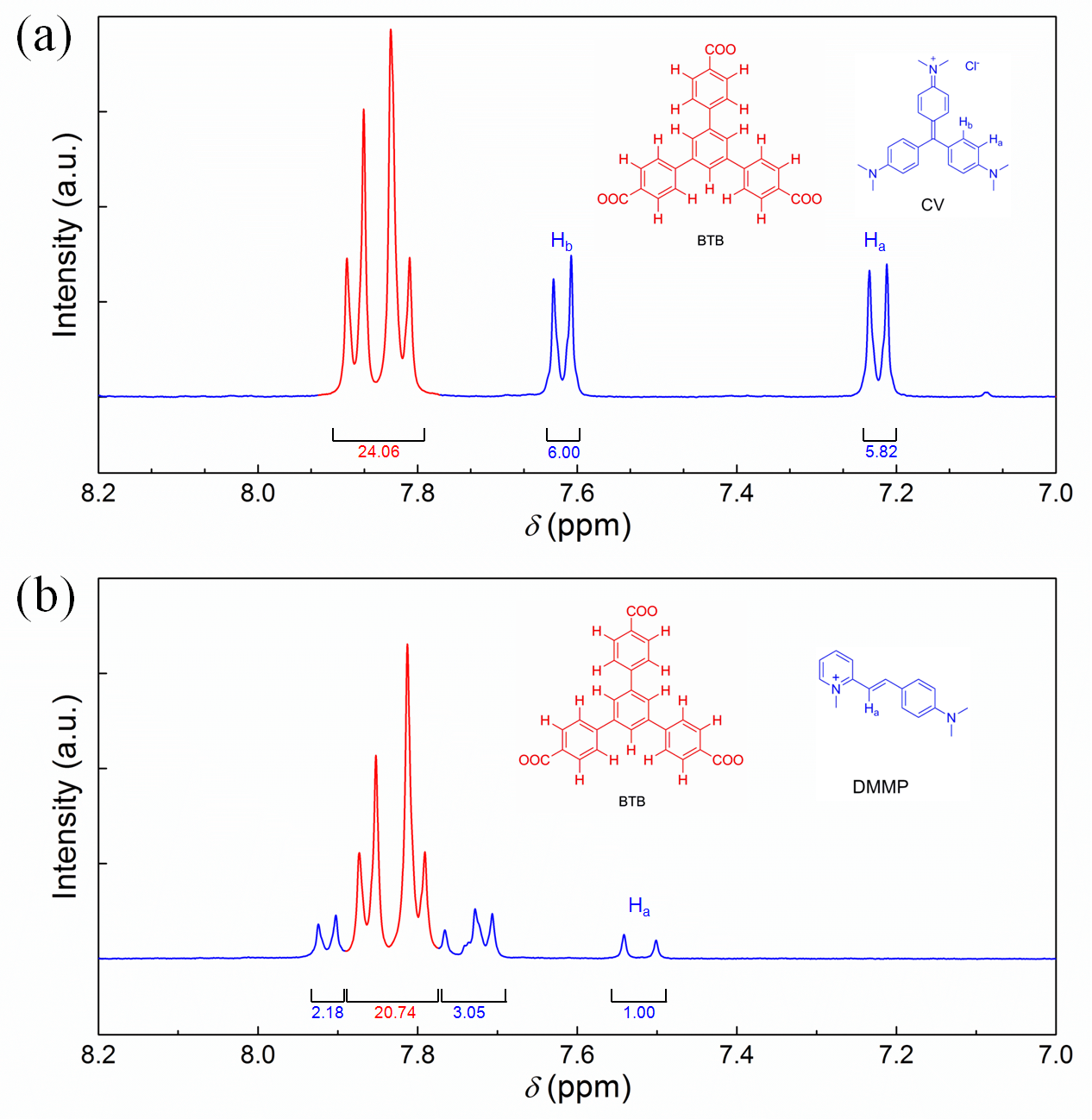
**

**Figure S15**. ^1^H-NMR spectra of the digested (a) CV@In-BTB and (b) DMMP@In-BTB in the mixture of DCl and DMSO-*d*_6_ (3:7 v/v%). The red signals indicate the resonance signals from BTB linker and the blue signals from CV and DMMP.

**
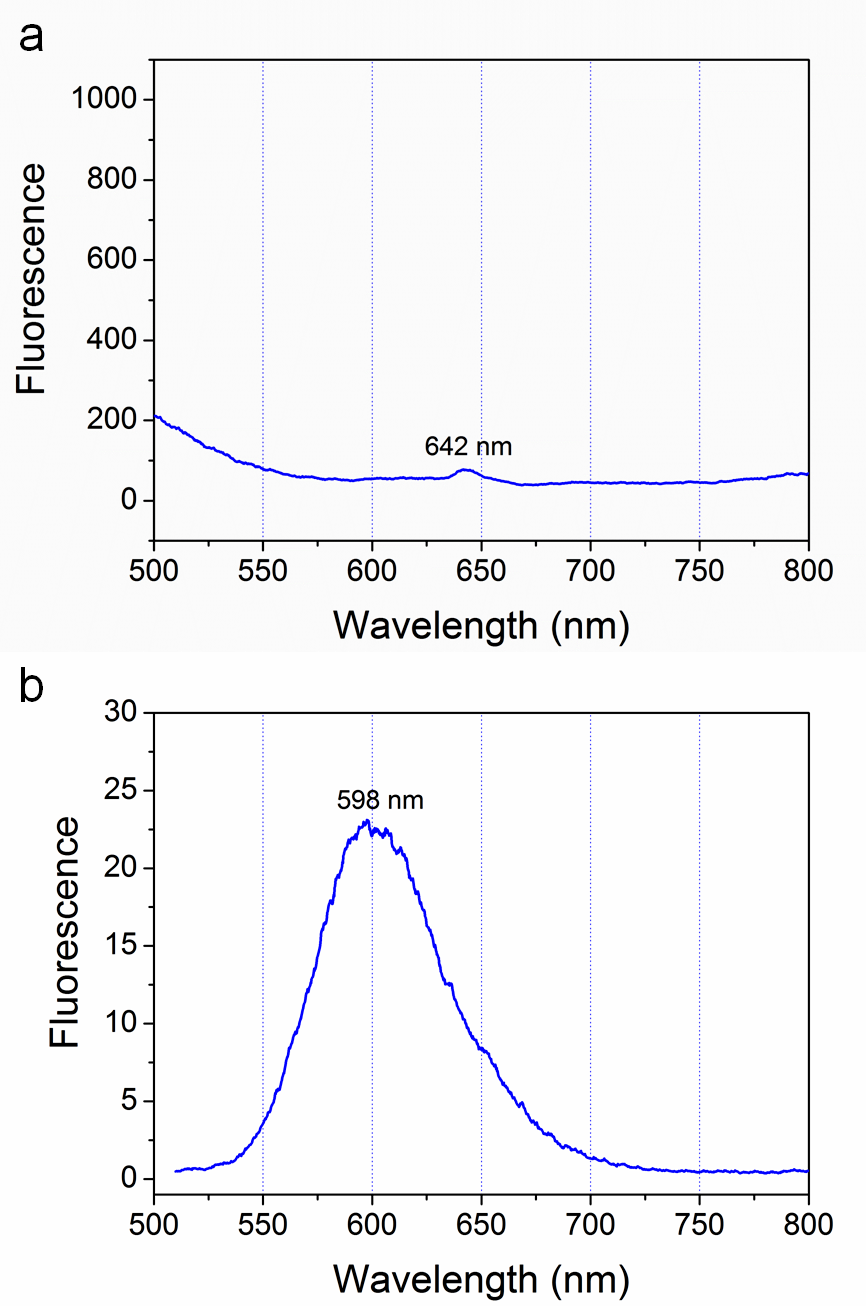
**

**Figure S16**. (a) Emission spectrum of NBA@In-BTB (λ_ex_ = 430 nm). (b) Emission spectrum of 1 × 10^-6^ M ethanolic solution of NBA (λ_ex_ = 500 nm).

**
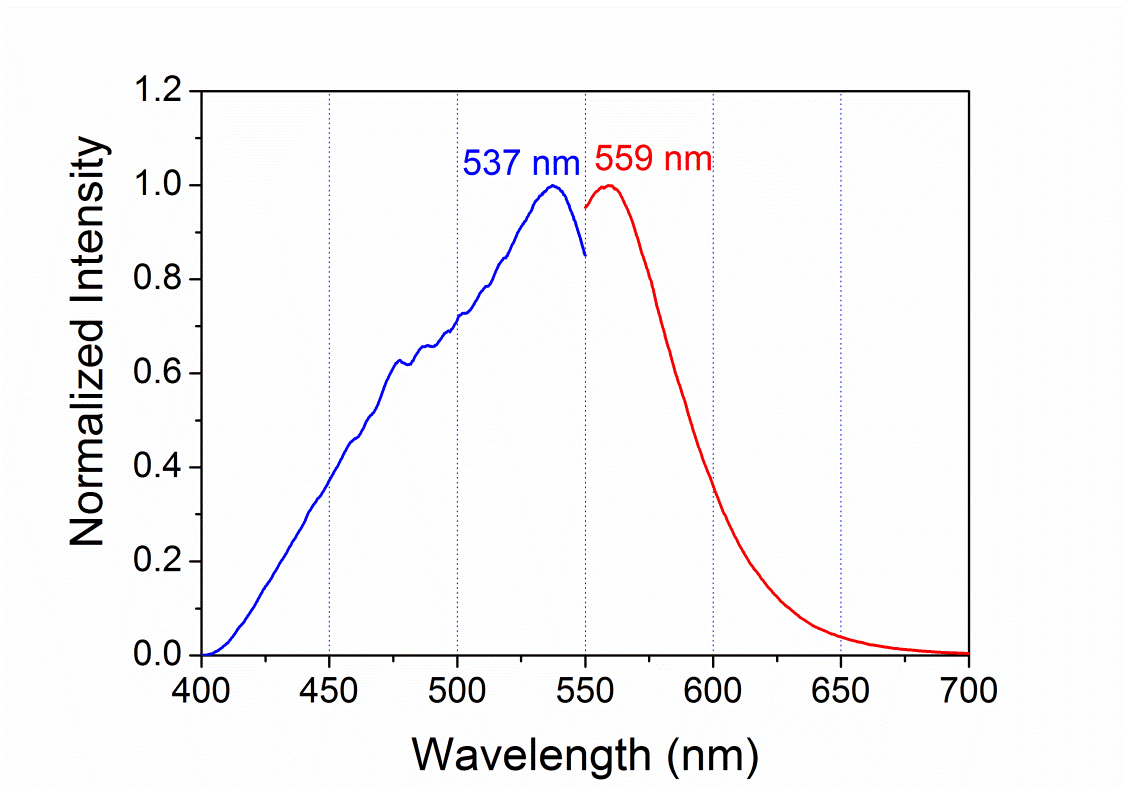
**

**Figure S17**. Normalized excitation (blue, λ_em_ = 560 nm) and emission (red, λ_ex_ = 537 nm) spectra of AOG@In-BTB.

**
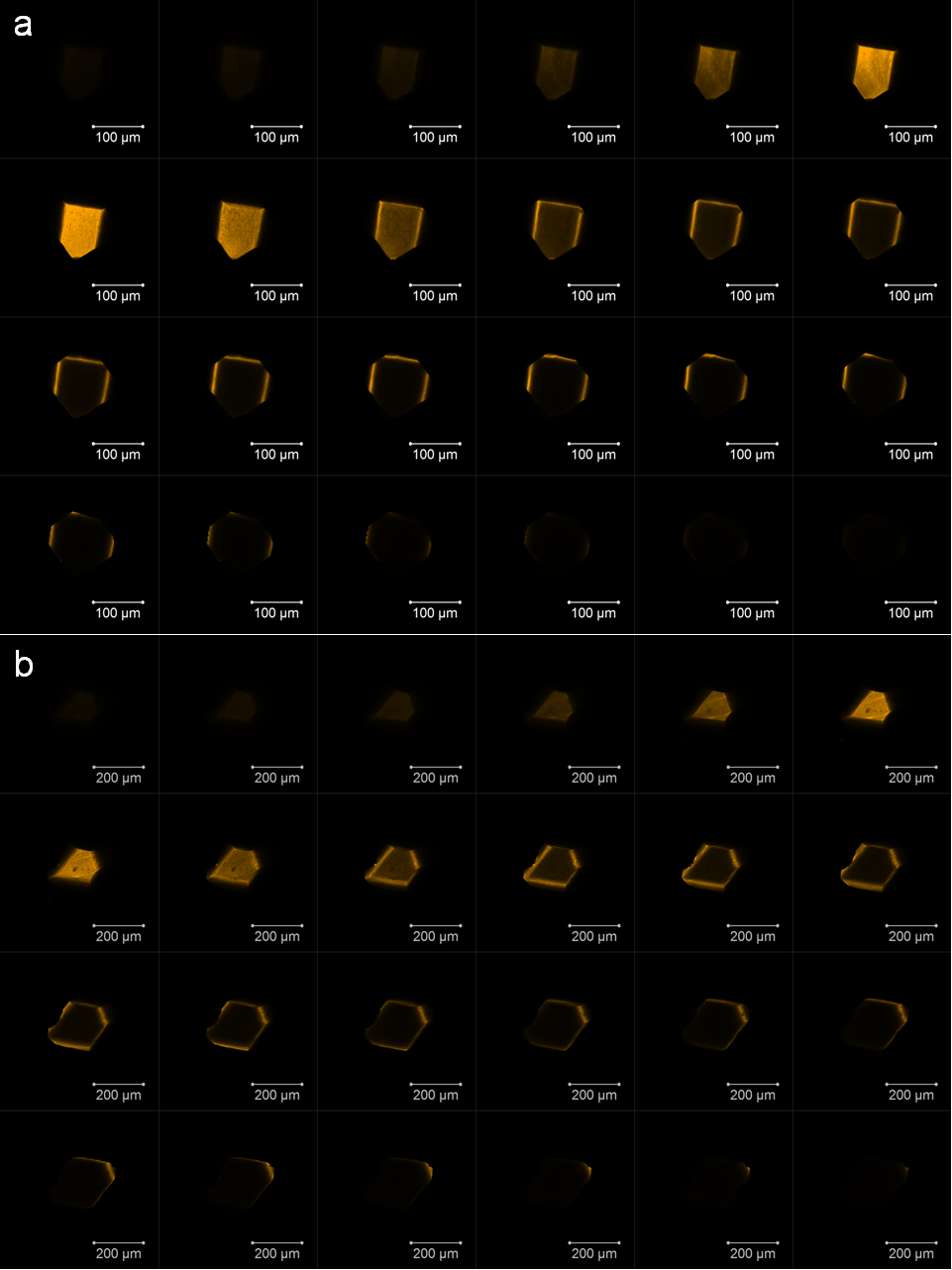
**

**Figure S18**. Z-stack CLSM images of AOG@In-BTB (λ_ex_ = 555 nm, λ_em_ = 585 nm). (a, b) Two different crystals were investigated.

**
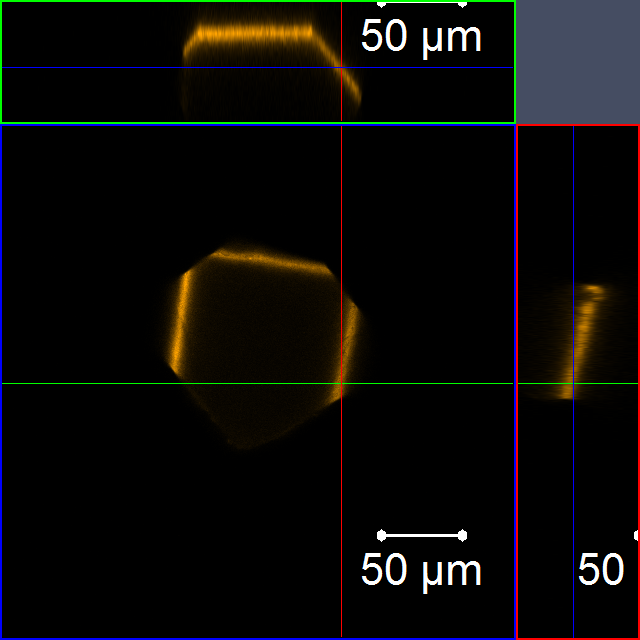
**

**Figure S19**. A sliced CLSM image of AOG@In-BTB (λ_ex_ = 555 nm, λ_em_ = 585 nm).

**Table S1**. Unit cell parameters of dye@In-BTBs including the relative differences from the original as-prepared In-BTB.

|  | **In-BTB** | **Rh6G@In-BTB** | | **NBA@In-BTB** | | **AOG@In-BTB** | |
| --- | --- | --- | --- | --- | --- | --- | --- |
|  |  |  | Relative difference |  | Relative difference |  | Relative difference |
| ***a*** | 44.2269(19) | 44.6694(10) | +0.4425 | 45.0162(8) | +0.7893 | 44.8184(13) | +0.5915 |
| ***b*** | 44.2269(19) | 44.6694(10) | +0.4425 | 45.0162(8) | +0.7893 | 44.8184(13) | +0.5915 |
| ***c*** | 42.519(2) | 41.477(11) | -1.0419 | 41.8896(9) | -0.6294 | 42.1828(14) | -0.3362 |
|  |  | **CV@In-BTB** | | **DEOCy@In-BTB** | | **DMMP@In-BTB** | |
|  |  |  | Relative difference |  | Relative difference |  | Relative difference |
| ***a*** |  | 45.2799(19) | +1.053 | 44.8708(7) | +0.6439 | 44.9141(10) | +0.6872 |
| ***b*** |  | 45.2799(19) | +1.053 | 44.8708(7) | +0.6439 | 44.9141(10) | +0.6872 |
| ***c*** |  | 41.588(2) | -0.931 | 42.0747(7) | -0.4443 | 41.9851(10) | -0.5339 |

**Table S2**. Encapsulation amounts of dyes by In-BTB measured by two different methods.

|  | UV/Vis spectroscopy  (mmol/mmol-In-BTB) | ^1^H-NMR spectroscopy  (mmol/mmol-In-BTB) |
| --- | --- | --- |
| **Rh6G@In-BTB** | 2.39  (0.321 mg/mg-In-BTB) | 2.50  (0.336 mg/mg-In-BTB) |
| **NBA@In-BTB** | 3.11  (0.319 mg/mg-In-BTB) | N.A. |
| **AOG@In-BTB** | N.A. | 1.08  (0.106 mg/mg-In-BTB) |
| **CV@In-BTB** | N.A. | 2.50  (0.286 mg/mg-In-BTB) |
| **DEOCy@In-BTB** | 2.36  (0.304 mg/mg-In-BTB) | N.A. |
| **DMMP@In-BTB** | 2.43  (0.249 mg/mg-In-BTB) | 2.86  (0.293 mg/mg-In-BTB) |
